# Adolescent Victimization and Early-Adult Psychopathology: Approaching Causal Inference Using a Longitudinal Twin Study to Rule Out Noncausal Explanations

**DOI:** 10.1177/2167702617741381

**Published:** 2017-12-12

**Authors:** Jonathan D. Schaefer, Terrie E. Moffitt, Louise Arseneault, Andrea Danese, Helen L. Fisher, Renate Houts, Margaret A. Sheridan, Jasmin Wertz, Avshalom Caspi

**Affiliations:** 1 Department of Psychology and Neuroscience, Duke University; 2 Center for Genomic and Computational Biology, Duke University; 3 Department of Psychiatry and Behavioral Sciences, Duke University; 4 Social, Genetic, and Developmental Psychiatry Centre, Institute of Psychiatry, Psychology, & Neuroscience, King’s College London; 5 Department of Child and Adolescent Psychiatry, Institute of Psychiatry, Psychology, and Neuroscience, King’s College London; 6 National and Specialist Child Traumatic Stress and Anxiety Clinic, South London and Maudsley National Health Service Foundation Trust, London, United Kingdom; 7 Department of Psychology and Neuroscience, The University of North Carolina, Chapel Hill

**Keywords:** victimization, adolescence, developmental psychopathology

## Abstract

Adolescence is the peak age for both victimization and mental disorder onset. Previous research has reported associations between victimization exposure and many psychiatric conditions. However, causality remains controversial. Within the Environmental Risk Longitudinal Twin Study, we tested whether seven types of adolescent victimization increased risk of multiple psychiatric conditions and approached causal inference by systematically ruling out noncausal explanations. Longitudinal within-individual analyses showed that victimization was followed by increased mental health problems over a childhood baseline of emotional/behavioral problems. Discordant-twin analyses showed that victimization increased risk of mental health problems independent of family background and genetic risk. Both childhood and adolescent victimization made unique contributions to risk. Victimization predicted heightened generalized liability (the “p factor”) to multiple psychiatric spectra, including internalizing, externalizing, and thought disorders. Results recommend violence reduction and identification and treatment of adolescent victims to reduce psychiatric burden.

Few problems in the psychological sciences have been as simultaneously important and intractable as establishing a causal relationship between victimization exposure and psychopathology. Because it is ethically impermissible to randomly assign human participants to varying levels of victimization exposure, observational studies have struggled to disentangle the effects of victimization exposure from a host of other individual and environmental factors (e.g., poverty, parent mental illness) known to be correlated with such exposure. Approaches using nonhuman models are likewise complicated by the fact that although experimenters can more easily control the level of exposure to stressful events in organisms like rodents and primates, the nonhuman analogues of victimization and psychopathology remain significantly divorced from their human counterparts, making it difficult to conclude that the results of these studies will generalize to the human condition.

Despite these challenges, studies reporting robust associations between victimization and various forms of psychopathology have continued to accumulate. According to this literature, exposure to victimization and other adverse life events (measured either retrospectively or prospectively) predicts increased risk of a wide array of psychiatric conditions, including mood, anxiety, substance use, disruptive behavior, and psychotic disorders (Anda et al., 2006; Green et al., 2010; Scott, Smith, & Ellis, 2010). Victimization exposure also predicts earlier onset, higher comorbidity, and greater numbers of symptoms among individual disorders, as well as poorer response to both pharmaceutical treatment and psychotherapy (Agnew-Blais & Danese, 2016; Nanni, Uher, & Danese, 2012; Nemeroff, 2016; Putnam, Harris, & Putnam, 2013; Widom, DuMont, & Czaja, 2007), leading some investigators to suggest that disorders arising after a history of victimization form their own clinically and biologically distinct subtype (Teicher & Samson, 2013).

Nevertheless, our understanding of the relationship between victimization and later mental health is characterized by at least four important gaps. First, it is difficult to determine whether observed associations between individual types of victimization and psychopathology reflect direct effects or arise solely as a result of the high rates of poly-victimization (i.e., exposure to multiple different types of victimization) among victimized children (Finkelhor, Ormrod, & Turner, 2007a, 2009). In other words, it is possible that the statistical association between exposure to one victimization type (e.g., family violence) and mental disorder exists solely because of one or more additional types of exposure associated with the exposure of initial interest (e.g., physical or sexual abuse). This “third variable problem” is significant because it limits the ability of researchers and policymakers to determine whether interventions that target a specific type of victimization will actually reduce the incidence of mental disorder. A potential solution is to ascertain multiple victimization types within the same sample (e.g., Fisher et al., 2015). This design allows investigators to examine both the shared and unique effects of different victimization types as well as the cumulative effects of poly-victimization.

A second limitation of the literature on victimization exposure and psychopathology is that previous studies have tended to focus on establishing associations between victimization exposure and an individual disorder. However, an emerging body of research indicates that the effects of victimization are strikingly nonspecific, predicting a wide range of both internalizing and externalizing symptoms (Edwards, Holden, Felitti, & Anda, 2003; Green et al., 2010; Putnam et al., 2013; Scott et al., 2010; Vachon, Krueger, Rogosch, & Cicchetti, 2015). Indeed, one study that examined associations between child maltreatment and multiple psychiatric disorders found that the effects of child maltreatment on mental health were mediated entirely through latent factors representing internalizing and externalizing psychopathology rather than diverse, specific mechanisms (Keyes et al., 2012). These findings suggest that maltreatment influences broad, general factors common to multiple different types of disorders (e.g., distress, negative emotionality) rather than those that give rise to specific disorders or clusters of symptoms.

One latent liability dimension that may be particularly suitable for testing the relationship between victimization exposure and later mental health is the “p factor,” a hierarchical measure of general psychopathology that accounts for the high levels of comorbidity observed across different psychiatric disorders. Conceptually similar to the “g factor” of general intelligence, “p” represents shared liability common to mental disorders captured by the internalizing, externalizing, and thought disorder spectra of psychopathology (Caspi et al., 2014; Lahey et al., 2012; Lahey, Krueger, Rathouz, Waldman, & Zald, 2017). Computation of a general factor of psychopathology thus allows investigators to examine associations between victimization exposure and broad vulnerability to multiple common mental disorders, whereas computation of its constituent psychiatric spectra permits testing for specificity in these associations (e.g., examining whether the mental health effects of victimization exposure are stronger for particular psychiatric spectra).

A third limitation of the existing literature on victimization exposure and psychopathology is that most of the research on the mental health effects of victimization has focused on childhood exposures. It is important to complement this literature with studies of adolescent exposures for two reasons. First, accumulating evidence demonstrates that adolescence is a crucial period of brain development as well as a time of peak onset for many common mental disorders (Kessler et al., 2005; Kim-Cohen et al., 2003). These findings have led to calls for research that will enhance our understanding of how experiences in adolescence contribute to disorders in adulthood (Davidson, Grigorenko, Boivin, Rapa, & Stein, 2015). Experimental and neuroimaging studies suggest that the increased incidence of psychopathology in adolescence may be partially attributable to the elevated stress reactivity and impaired extinction learning that emerge during this period (Pattwell et al., 2012; Spear, 2009) as well as the lagged development of cortical regions that play a key role in emotion regulation (e.g., the prefrontal cortex; Gogtay et al., 2004). Combined, these findings suggest that exposure to victimization during adolescence may be associated with a physiological response that is both larger in magnitude and more difficult to downregulate than an equivalent exposure in childhood, perhaps leading to a relatively stronger relationship between victimization during the adolescent period and the development of psychiatric symptoms. However, the relative contribution of victimization experiences in childhood versus those in adolescence has rarely been tested in one sample.

Another reason to study victimization in adolescence is that exposure to many types of victimization—including sexual victimization, relational aggression, Internet harassment, and serious violent crime—also peaks during this period (Brown, Birch, & Kancherla, 2005; Peskin, Tortolero, & Markham, 2006; Sickmund & Puzzanchera, 2014). Because of the increased autonomy and greater Internet and cell phone use that characterize the adolescent period, adolescents are, on average, victimized by a more diverse set of actors and across a wider range of environments than children (Sickmund & Puzzanchera, 2014). Moreover, most victimization experiences in childhood are shared by siblings, especially twins (Jaffee et al., 2004), making it difficult to assess whether victimization exposure exerts an environmentally mediated effect on mental health using a discordant-twin design. In adolescence, however, exposure to victimization becomes more divergent as members of twin pairs spend more time apart and outside of the shared family environment with increasing age, making this analytical approach significantly more viable.

A fourth limitation of the existing literature on victimization exposure and psychopathology is the elephant in the room: Is the intuitive assumption that exposure to victimization exerts a causal effect on later mental health validated by empirical data (Moffitt & the Klaus-Grawe ThinkTank, 2013)? Although causality cannot be proven by observational studies, these designs can allow researchers to rule out alternate, noncausal explanations, making the existence of a causal relationship incrementally more likely. One of the strongest observational designs for approaching causal inference in this fashion is the longitudinal twin study, which allows investigators to control for all of the unmeasured shared environmental or genetic factors that might impact both the exposure and the outcome of interest. To date, however, twin studies conducted using twin pairs discordant for victimization exposure have returned conflicting results, with some studies reporting an increased risk of emotional or behavioral problems in the more-victimized twin (Arseneault et al., 2011; Arseneault et al., 2006; Brown et al., 2014; Capusan et al., 2016; Kendler & Aggen, 2014; Silberg et al., 2016), and others reporting little to no effect (Berenz et al., 2013; Bornovalova et al., 2013; Dinkler et al., 2017; Dinwiddie et al., 2000; Shakoor et al., 2015; Young-Wolff, Kendler, Ericson, & Prescott, 2011). These studies are particularly difficult to reconcile because they studied different victimization types and different disorders in different populations assessed at different ages.

We used data from a longitudinal twin study (the Environmental Risk Longitudinal Twin Study [E-Risk]), in which we have ascertained multiple forms of victimization, to test associations between adolescent victimization exposure and multiple forms of psychopathology (internalizing, externalizing, and thought disorders), including a general liability factor (“p”; Caspi et al., 2014; Lahey et al., 2012; Lahey et al., 2017). In conducting such tests, we extend previous work, which examined a limited range of exposures (most often victimization by family members, including physical maltreatment, neglect, or sexual abuse), to examine a larger range of exposures occurring both inside and outside the home (e.g., peer victimization, Internet/mobile phone victimization, exposure to conventional crime). We also used a cumulative measure of poly-victimization between ages 12 and 18 years. We examined the specificity of effects in our data, testing (a) whether each separate form of victimization uniquely predicts early-adult psychopathology and (b) whether victimization exposure predicts some forms of psychopathology more strongly than others. We then carried out four analyses aimed at approaching causal inference by ruling out noncausal explanations. First, we tested for mono-method reporting bias—or the possibility that the association between victimization exposure and early-life psychopathology exists solely because both rely on self-report data—by examining whether “p” can also be predicted by informant-reported victimization exposure, provided by E-Risk members’ parents and co-twins. Second, we addressed the possibility of reverse causation by testing whether adolescent victimization predicts “p” only because children with preexisting vulnerabilities to psychiatric problems (such as early-life emotional and behavioral problems or a family history of mental disorder) are more likely to be victimized. Third, we tested whether adolescent victimization makes its own contribution to psychopathology apart from the contribution of child victimization (i.e., revictimization). Fourth, we exploited our twin study design to test whether the observed relationship between victimization and psychopathology is attributable to shared genetic propensity, shared family-wide environmental factors (e.g., family poverty), and preexisting differences between twins in their vulnerability to later psychopathology.

## Method

### Study sample

Participants were members of E-Risk, which tracks the development of a birth cohort of 2,232 British children. The sample was drawn from a larger birth register of twins born in England and Wales in 1994–1995 (Trouton, Spinath, & Plomin, 2002). Full details about the sample are reported elsewhere (Moffitt & the E-Risk Study Team, 2002). In brief, the E-Risk sample was constructed in 1999–2000, when 1,116 families (93% of those eligible) with same-sex 5-year-old twins participated in home-visit assessments. This sample comprised 56% monozygotic (MZ) and 44% dizygotic (DZ) twin pairs; sex was evenly distributed within zygosity (49% male). Of the full sample, 7% self-identified as Black, Asian, or mixed race. Families were recruited to represent the U.K. population with newborns in the 1990s on the basis of maternal age and geographic location to both ensure adequate numbers of children in disadvantaged homes and avoid an excess of twins born to well-educated women using assisted reproduction. The study sample represents the full range of socioeconomic conditions in Great Britain, as reflected in the families’ distribution on a neighborhood-level socioeconomic index (ACORN [A Classification Of Residential Neighborhoods], developed by CACI Inc. for commercial use; Odgers, Caspi, Bates, Sampson, & Moffitt, 2012): 25.6% of E-Risk families live in “wealthy achiever” neighborhoods compared with 25.3% nationwide; 5.3% versus 11.6% live in “urban prosperity” neighborhoods; 29.6% versus 26.9% live in “comfortably off” neighborhoods; 13.4% versus 13.9% live in “moderate means” neighborhoods; and 26.1% versus 20.7% live in “hard-pressed” neighborhoods. E-Risk underrepresents urban prosperity neighborhoods because such households are likely to be childless.

Follow-up home visits were conducted when participants were ages 7 (98% participation), 10 (96% participation), 12 (96% participation), and most recent, 18 (93% participation) years. At age 18 years, 2,066 participants were assessed, each twin by a different interviewer. The average age at the time of assessment was 18.4 years (*SD* = 0.36); all interviews were conducted after the 18th birthday. There were no differences between those who did and did not take part at age 18 years in terms of socioeconomic status (SES) assessed when the cohort was initially defined (χ^2^ = 0.86, *p* = .65), age-5 IQ scores (*t* = 0.98, *p* = .33), age-5 internalizing or externalizing behavior problems (*t* = 0.40, *p* = .69 and *t* = 0.41, *p* = .68, respectively), or childhood poly-victimization (*z* = 0.51, *p* = .61).

The Joint South London, Maudsley, and Institute of Psychiatry Research Ethics Committee approved each phase of the study. Parents gave informed consent and twins gave assent between 5 and 12 years old and then informed consent at age 18 years.

### Measures

The remainder of the Method section is divided into four parts. Part I describes the measurement of victimization across the study participants’ first two decades of life (birth to age 18 years). Part II describes the measurement of psychiatric symptoms at age 18 years. Part III describes our creation of factor scores for the internalizing, externalizing, and thought disorder spectra, as well as for the “p factor,” corresponding to E-Risk members’ general liability to psychopathology at age 18 years. Part IV describes covariates used in our analyses. The design of the sample and data for this article are diagrammed in Figure S1 in the Supplemental Material available online.

#### Part I. Assessment of victimization exposure

##### Childhood victimization

These measures have been described previously (Danese et al., 2017; details are provided in the Supplemental Material). In brief, exposure to several types of victimization was assessed repeatedly when the children were 5, 7, 10, and 12 years of age. These were exposure to domestic violence between the mother and her partner, frequent bullying by peers, physical maltreatment by an adult, sexual abuse, emotional abuse and neglect, and physical neglect. Exposure to each type of victimization was coded on a 3-point scale, in which 0 indicated *no exposure*, 1 indicated *probable* and *less severe* exposure, and 2 indicated *definite* and *severe* exposure.

##### Childhood poly-victimization

We study poly-victimization because previous studies have indicated that it is a considerably more powerful predictor of psychiatric symptoms than the presence or absence of any particular exposure, with poly-victimized children tending to experience more symptoms than even children who were repeatedly exposed to one kind of victimization experience (Finkelhor et al., 2007a). Following Finkelhor et al. (2007a), we used the most straightforward and reproducible method to define poly-victimization, operationalized as the simple count of forms of victimization experienced by a child (exposure to domestic violence between the mother and her partner, frequent bullying by peers, physical maltreatment by an adult, sexual abuse, emotional abuse and neglect, and physical neglect). This variable was derived by summing all childhood victimization experiences coded as 2: 1,641 (73.5%) of children had zero severe victimization experiences; 448 (20.1%) had one; 85 (3.8%) had two; 39 (1.8%) had three; 17 (0.8%) had four; and 2 (0.1%) had five severe victimization experiences. Next, we winsorized the poly-victimization distribution into a four-category variable (representing 0, 1, 2, and 3+ severe experiences). In addition, we conducted a sensitivity test by analyzing the data using both the winsorized and nonwinsorized exposure variables, and we observed the same results.

##### Adolescent victimization

These measures have been described previously (Fisher et al., 2015; details are provided in the Supplemental Material). In brief, participants were interviewed at age 18 about exposure to a range of adverse experiences between 12 and 18 years using the Juvenile Victimization Questionnaire, 2nd revision (JVQ; Finkelhor, Hamby, Turner, & Ormrod, 2011; Hamby, Finkelhor, Ormrod, & Turner, 2004), adapted as a clinical interview. Each co-twin was interviewed by a different research worker, and each JVQ question was asked for the period “since you were 12.” Age 12 is a salient age for our participants because it is the age when British children leave primary school to enter secondary school. The JVQ has good psychometric properties (Finkelhor, Hamby, Ormrod, & Turner, 2005) and was used in the U.K. National Society for the Prevention of Cruelty to Children national survey (Radford et al., 2011; Radford, Corral, Bradley, & Fisher, 2013), thereby providing important benchmark values for comparisons with our cohort. Our adapted JVQ comprised 45 questions covering seven different forms of victimization: maltreatment, neglect, sexual victimization, family violence, peer/sibling victimization, Internet/mobile phone victimization, and crime victimization. Like childhood victimization, exposure to each type of adolescent victimization was also coded on a 3-point scale, in which 0 indicated *no exposure*, 1 indicated *probable* or *less severe* exposure, and 2 indicated *definite* or *severe* exposure.

The adolescent poly-victimization variable was derived by summing all victimization experiences that received a code of 2 (i.e., severe exposure): 1,332 (64.6%) of adolescents had zero severe victimization experiences; 396 (19.2%) had one; 195 (9.5%) had two; 93 (4.5%) had three; 30 (1.5%) had four; 11 (0.5%) had five; and 5 (0.2%) had six severe victimization experiences. Poly-victimization is common among adolescents in our sample; of E-Risk members who experienced at least one type of severe victimization, nearly half (46%) also reported exposure to multiple different types of victimization.

As with childhood victimization, we winsorized the adolescent poly-victimization distribution into a four-category variable (0, 1, 2, and 3+ severe experiences). In addition, we conducted a sensitivity test by analyzing the data using both the winsorized and nonwinsorized exposure variables, and we observed the same results.

##### Informant reports of adolescent victimization

At age 18, each E-Risk member’s co-twin and parent (usually mother) were asked to reply to a confidential questionnaire that used a seven-item checklist to inquire whether the E-Risk member had ever been the victim of each of the seven different forms of victimization included in the JVQ interview: maltreatment, neglect, sexual abuse, exposure to family violence, peer bullying, Internet harassment, or a violent crime. We summed affirmative responses to these questions, within each reporter. The correlation between co-twin and parental reports was *r* = .38; between co-twin and E-Risk members’ JVQ reports, *r* = .38; and between parental and E-Risk members’ JVQ reports, *r* = .34.

#### Part II. Assessment of symptoms of mental disorders

At age 18, E-Risk members were assessed in private interviews about symptoms of mental disorders (see Table S1 in the Supplemental Material). We assessed past-year symptoms of five externalizing-spectrum disorders: *Diagnostic and Statistical Manual of Mental Disorders* (4th ed.; *DSM–IV*; American Psychiatric Association, 1994) symptoms of alcohol dependence and cannabis dependence were assessed via the Diagnostic Interview Schedule (Robins, Cottler, Bucholz, & Compton, 1995); conduct disorder was measured by inquiring about *DSM–IV* symptoms (American Psychiatric Association, 1994); symptoms of tobacco dependence were assessed with the Fagerstrom Test for Nicotine Dependence (Heatherton, Kozlowski, Frecker, & Fagerström, 1991); and attention-deficit/hyperactivity disorder (ADHD) was measured by inquiring about *Diagnostic and Statistical Manual of Mental Disorders* (5th ed.; *DSM–5*; American Psychiatric Association, 2013) symptoms (Agnew-Blais et al., 2016). We also assessed past-year symptoms of four internalizing-spectrum disorders: *DSM–IV* symptoms of depression, generalized anxiety disorder, and posttraumatic stress disorder (PTSD) were assessed via the Diagnostic Interview Schedule (Robins et al., 1995), and symptoms of eating disorder were assessed with the SCOFF (Morgan, Reid, & Lacey, 1999). We assessed symptoms of thought disorder in two ways: First, each E-Risk member was interviewed about delusions and hallucinations (e.g., “Have other people ever read your thoughts?”; “Have you ever thought you were being followed or spied on?”; “Have you ever heard voices that other people cannot hear?”). This interview was also administered at an earlier age to E-Risk members and its scoring system is described in detail elsewhere (Polanczyk et al., 2010b). Second, each E-Risk member was asked about unusual thoughts and feelings (e.g., “My thinking is unusual or frightening”; “People or places I know seem different”), drawing on item pools since formalized in prodromal psychosis instruments, including the PRIME-screen and SIPS (Loewy, Pearson, Vinogradov, Bearden, & Cannon, 2011).

#### Part III. The structure of psychopathology at age 18

Using confirmatory factor analysis, we tested two standard models (Brunner, Nagy, & Wilhelm, 2012; Rindskopf & Rose, 1988) that are frequently used to examine hierarchically structured constructs: a correlated-factors model with three factors (representing internalizing, externalizing, and thought disorders; see Fig. S2a in the Supplemental Material) and a bi-factor model specifying a general psychopathology factor (labeled “p”; see Fig. S2b in the Supplemental Material). (The use of the term *bi-factor model* is an unwieldy historical and statistical necessity; it harkens back to the early days of psychometric research on intelligence, which first proposed a general factor that is common to all items on a test and more specific factors that are common to a smaller subset of related items.) Both the correlated-factors and bi-factor models included the 11 observed variables described in Part II of the Measures section (i.e., alcohol dependence, cannabis dependence, tobacco dependence, conduct disorder, ADHD, anxiety, depression, eating disorders, PTSD, psychotic-like experiences, prodromal symptoms). We were guided in decisions regarding which disorders loaded on which factors by the Hierarchical Taxonomy of Psychopathology consortium (https://medicine.stonybrookmedicine.edu/HITOP/AboutHiTOP; Kotov et al., 2017). As such, symptoms corresponding to disorders of substance use (i.e., alcohol, marijuana, smoking) and oppositional behavior (i.e., conduct disorder and ADHD) loaded on the externalizing factor; symptoms corresponding to disorders of distress (i.e., major depressive episode, generalized anxiety disorder, and PTSD) and eating pathology (i.e., eating disorder) loaded on the internalizing factor; and symptoms corresponding to disorders associated with psychosis loaded on the thought disorder factor. Details concerning model fit, factor loadings, and comparisons are presented in Tables S2 to S4 in the Supplemental Material.

Although both the correlated-factors model and bi-factor model fit the data well, the bi-factor model proved to be a better fit, consistent with the notion of a general factor of psychopathology (“p”). We present results from both the correlated-factors and bi-factor models because both feature prominently in the literature. Presenting both models enables us to address questions of specificity and test whether the “p factor” might offer a more parsimonious account of any nonspecificity observed using the factors representing internalizing, externalizing, and thought disorder symptoms from the correlated-factors model. We calculate “p” using the bi-factor model because it is the most commonly reported general factor model in the existing literature (Caspi et al., 2014; Greene & Eaton, 2017; Laceulle, Vollebergh, & Ormel, 2015; Lahey et al., 2012; Lahey et al., 2015; Martel et al., 2017; Murray, Eisner, & Ribeaud, 2016; Olino, Dougherty, Bufferd, Carlson, & Klein, 2014; Patalay et al., 2015; Snyder, Young, & Hankin, 2017b). For expository purposes, we scaled E-Risk members’ scores on each factor to a mean of 100 and standard deviation of 15.

#### Part IV. Covariates

##### Mental health and substance problems in early adolescence (age 12)

We assessed seven different signs of mental health difficulties at age 12. These were summed to create an index of the number of different types of early-adolescent mental health problems, ranging from 0 to 7. As previously described (e.g., Polanczyk et al., 2010a), ADHD and conduct disorder were ascertained using *DSM–IV* criteria on the basis of mother and teacher reports of symptoms shown within the past 6 months. Clinically significant anxiety was considered present if children scored above the 95th percentile (score ≥ 13) on the 10-item Multidimensional Anxiety Scale for Children (March, Parker, Sullivan, Stallings, & Conners, 1997). Clinically significant depression was considered present if children scored ≥ 20 on the Children’s Depression Inventory (Kovacs, 1992). Children were considered to engage in harmful substance use if they reported that they had tried drinking alcohol or smoking cigarettes on more than two occasions or had tried cannabis, taken pills to get high, or sniffed glue/gas on at least one occasion. Children were coded as having engaged in self-harm/suicidal behavior if the primary caregiver reported that the child had deliberately harmed himself or herself or attempted suicide in the previous 6 months (Fisher et al., 2012). (We asked only mothers to report at this age because of ethical considerations.) We ascertained the presence of psychotic symptoms in a private interview conducted with the children (Polanczyk et al., 2010b). Our protocol took a conservative approach to designating a child’s report as a symptom. (a) When a child endorsed any symptom, the interviewer probed using standard prompts designed to discriminate between experiences that were plausibly real (e.g., “I was followed by a man after school”) and potential symptoms (e.g., “I was followed by an angel who guards my spirit”). (b) Two psychiatrists and a psychologist reviewed all written narratives to confirm the codes (but without consulting other data sources about the child or family). (c) Because ours was a sample of twins, experiences limited to the twin relationship (e.g., “My twin and I often know what each other is thinking”) were coded as “not a symptom.”

##### Emotional and behavioral problems in early childhood (age 5)

We assessed internalizing and externalizing problems at age 5 by using the Child Behavior Checklist in interviews with mothers and the Teacher Report Form by mail for teachers (Achenbach, 1991a, 1991b). The internalizing problems scale is the sum of items in the withdrawn and anxious/depressed subscales, and the externalizing problems scale is the sum of items from the aggressive and delinquent behavior subscales. We summed and standardized mothers’ and teachers’ reports of each of these measures to create a single cross-informant scale representing total emotional and behavioral problems.

##### Family history of psychiatric disorder

This was ascertained at the age-12 assessment from reports by biological parents conducted as part of a family history interview (Milne et al., 2008). Family history of psychiatric disorder was defined as a report of treatment or hospitalization for a psychiatric disorder or substance-use problem, or attempted or completed suicide for any of the child’s biological mother, father, grandparents, or aunts and uncles. We report the proportion of family members with any of these conditions.

## Results

### Does victimization in adolescence predict early-adult psychopathology?

We examined the extent to which adolescent victimization predicted early-adult psychopathology (“p”) using four sets of linear mixed models, which control for the clustering within families.

**Fig. 1.**
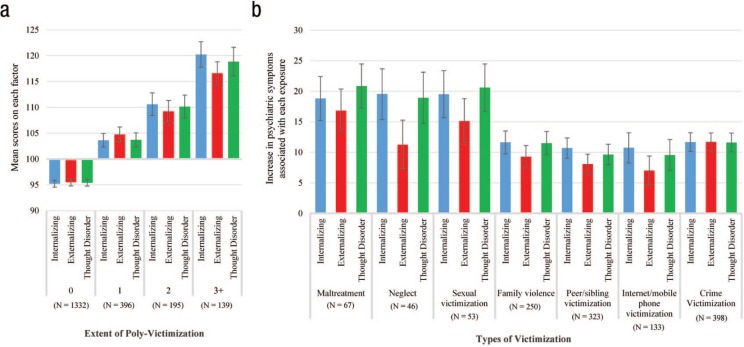
Associations between adolescent victimization and early-adult psychopathology, divided into its constituent symptom spectra (internalizing, externalizing, and thought disorders). (a) Mean scores on internalizing, externalizing, and thought disorders (from the correlated-factors model) at age 18 for Environmental Risk Longitudinal Twin Study (E-Risk) members exposed to 0, 1, 2, or 3+ types of severe adolescent victimization. All factor scores are scaled to a sample mean of 100 and a standard deviation of 15. (b) Estimates reflect coefficients from separate linear mixed models, which control for clustering by family. These coefficients represent the average difference in internalizing, externalizing, and thought disorder factor scores (from the correlated-factors model) at age 18 between exposed and nonexposed E-Risk members in standardized units, where 15 points equals 1 standard deviation. Error bars represent 95% confidence intervals.

First, we tested whether E-Risk members’ scores on each of the three factors (i.e., internalizing, externalizing, and thought disorders) from the correlated-factors model could be predicted by an omnibus measure of victimization exposure—adolescent poly-victimization. This measure reflects the number of different types of severe victimization experiences to which each E-Risk member had been exposed. As shown in Figure 1a, increasing levels of poly-victimization were associated with significant elevations across all three factor scores.

Second, we tested whether severe exposure to each individual type of victimization in adolescence (i.e., maltreatment, neglect, sexual victimization, family violence, peer/sibling victimization, Internet/mobile phone victimization, and crime victimization) was also associated with significant elevations across all three factor scores. We found that it was (see Fig. 1b). Importantly, the magnitude of these associations within each victimization type was also roughly similar across factors. This pattern suggests that all seven types of adolescent victimization have negative but largely nonspecific associations with early-adult mental health.

**Fig. 2.**
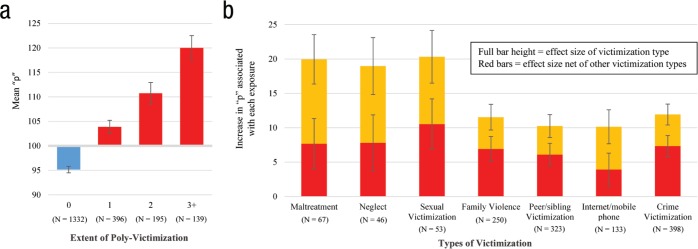
Associations between adolescent victimization exposure and early-adult psychopathology (“p”). (a) Mean scores on “p” for Environmental Risk Longitudinal Twin Study (E-Risk) members exposed to 0, 1, 2, or 3+ types of severe adolescent victimization. We scaled “p” to a sample mean of 100 and a standard deviation of 15. (b) Estimates here represent coefficients from separate and simultaneous linear mixed models, which control for clustering by family. These coefficients represent the average difference in “p” between exposed and nonexposed E-Risk members in standardized units where 15 points equals 1 standard deviation. The height of each full bar depicts the effect size of the association between exposure to each victimization type and “p” scores. The height of the red bars depicts the unique association between exposure to each victimization type and “p” scores, while controlling for exposure to each of the other six victimization types. *N*s reflect the number of E-Risk members who were exposed to severe forms of each victimization type. Error bars represent 95% confidence intervals. Exact values for these estimates, as well as corresponding 95% confidence intervals and *p* values, can be found in Table S5 in the Supplemental Material.

Third, we tested whether poly-victimization in adolescence predicted E-Risk members’ scores on “p” from the bi-factor model, a measure of general liability to multiple forms of psychopathology. In our cohort, poly-victimization during adolescence was positively associated with “p” (*b* = 7.74, *p* < .001), with each additional severe victimization type predicting an approximately 0.5 standard deviation increase (see Fig. 2a). This finding suggests that the nonspecific effects of victimization exposure on multiple psychiatric spectra are likely attributable to its association with this higher order general liability factor.

Fourth, we tested the predictive relationship between exposure to each victimization type and “p,” both separately and in a model in which all seven victimization types were entered simultaneously. These analyses allowed us to test whether each type of victimization was associated directly with “p,” independent of its co-occurrence with other forms of victimization. As shown by the full bars in Figure 2b, severe exposure to each individual type of adolescent victimization was significantly associated with increased “p” at age 18. We also observed significantly stronger effects for maltreatment, neglect, and sexual victimization relative to other victimization types. As shown by the shorter, red bars in Figure 2b, when the seven types of adolescent victimization were simultaneously entered to predict “p,” all remained significant, indicating that each exposure type exerted its own unique effect on “p.” In addition, the effects of maltreatment, neglect, and sexual victimization were significantly attenuated in this simultaneous model, bringing the effect estimates for severe maltreatment and neglect roughly in line with estimates for the other exposures (see Table S5 in the Supplemental Material for more detail). This attenuation suggests that the greater increases in “p” associated with these exposures are likely attributable to higher levels of poly-victimization also associated with these exposures.

We found no consistent pattern of sex differences in our sample. There was no significant gender interaction in the association between adolescent poly-victimization and early-adult “p” (*b* = 0.23, *p* = .722); the association between adolescent poly-victimization and early-adult “p” was comparable for males (*b* = 7.57, *p* < .001) and females (*b* = 7.87, *p* < .001). Similarly, only one significant gender interaction was noted in the relationship between each type of victimization and “p” at age 18; crime victimization had a slightly stronger association with “p” for females (*b* = 15.51, *p* < .001) than for males (*b* = 10.35, *p* < .001), *b*_interaction_ = 5.15, *p* = .001 (see Table S6 in the Supplemental Material).

### What accounts for the predictive relationship between adolescent victimization and “p”?

Although our results demonstrated that E-Risk members exposed to more victimization in adolescence tended to score higher on “p,” this statistical relationship could arise from one of several distinct, noncausal processes. We next describe and systematically test four of the most plausible noncausal explanations.

#### Is the relationship between adolescent victimization and “p” a spurious artifact of two single-source measures?

It is possible that the relationship between adolescent poly-victimization and “p” occurs only because both measures rely on self-report data, generating an inflated association as a result of shared method variance (Bank, Dishion, Skinner, & Patterson, 1990). For example, exclusive reliance on self-report measures raises the possibility that the higher levels of victimization exposure reported by participants with psychiatric symptoms may, in fact, reflect the effects of phenomena such as mood-congruent recall rather than greater exposure to such experiences per se (Reuben et al., 2016; Susser & Widom, 2012).

We tested this possibility by using a linear mixed model to predict “p” as a function of either self- or informant-reported victimization exposure during adolescence. If the relationship between poly-victimization and “p” were a result of self-report bias, we would expect to find a significant association between self-reported adolescent poly-victimization and “p” but little to no association between co-twin-reported or parent-reported victimization and “p.” Instead, however, we found both self- and informant-reported adolescent exposure to be significant predictors, with each additional type of parent-reported victimization (*b* = 5.64, *p* < .001) and co-twin-reported victimization (*b* = 5.14, *p* < .001) predicting an approximately 0.3 standard deviation increase in “p” (see Table S7 in the Supplemental Material). This pattern of results suggests that the observed association between self-reported adolescent victimization and “p” (*b* = 7.74, *p* < .001) cannot be explained solely by mono-method reporting biases. The effect size was smaller for informant reports, perhaps because they were collected via questionnaire checklists uncoded for severity, whereas self-reports were collected via clinical interviews and coded for severity.

#### Does adolescent victimization predict poorer early-adult mental health because preexisting psychiatric vulnerabilities increase the risk of victim-ization? (the “reverse causation” hypothesis)

If mental disorders are neurodevelopmental conditions that have their roots in early life, it is possible that E-Risk members’ psychiatric symptoms at age 18 were also present in childhood and that their higher levels of adolescent victimization exposure are a consequence of these symptoms. Rather than suggest a causal effect of adolescent victimization on “p,” this “reverse causation” explanation instead proposes that the statistical relationship between these two constructs arises because children with more mental health problems are more likely to be victimized when they enter adolescence.

We tested this possibility using two sets of linear mixed models. In the first set, we tested whether adolescent poly-victimization was predicted by each of three different measures of early-life vulnerability to adult psychiatric disorder. These three measures were (a) a count of mental health problems assessed at age 12, (b) a score representing parent- and teacher-reported emotional and behavioral problems at age 5, and (c) family history of psychiatric disorder. Our results indicated that higher scores on each type of childhood psychiatric vulnerability were associated with greater adolescent victimization exposure as well as higher scores on “p” (see Table 1).

**Table 1.** Associations Between Preexisting Psychiatric Vulnerabilities, Adolescent Victimization, and Early-Adult Psychopathology (“p”)

Preexisting psychiatric vulnerabilities (*z*-scored)	Adolescent poly-victimization (ages 12-18) (*z*-scored)				Early-adult psychopathology (age 18) (“p”; *M* = 100, *SD* = 15)			
	(1)	(2)	(3)	(4)	(1)	(2)	(3)	(4)
Mental health problems (age 12)	0.16**[0.11, 0.20]	—	—	0.14**[0.09, 0.18]	3.39**[2.73, 4.05]	—	—	2.88**[2.20, 3.55]
Emotional and behavioral problems (age 5)	—	0.11**[0.07, 0.16]	—	0.04[−0.01, 0.09]	—	2.48**[1.81, 3.15]	—	1.19*[0.46, 1.92]
Proportion of family members with any disorder	—	—	0.15**[0.09, 0.20]	0.11**[0.05, 0.15]	—	—	2.98**[2.23, 3.74]	2.14**[1.37, 2.90]

Note: In all linear mixed models, the three preexisting psychiatric vulnerabilities and adolescent poly-victimization were all standardized to a *z*-score with mean of 0 and a standard deviation of 1 to facilitate comparison across measures, whereas “p” remains scaled to a mean of 100 with a standard deviation of 15. 95% confidence intervals are reported in brackets.

* *p* < .01. ** *p* < .001.

Consequently, our next set of analyses tested whether adolescent poly-victimization predicted “p” above and beyond the effects associated with these preexisting psychiatric vulnerabilities. We conducted four linear mixed model regressions predicting “p” at age 18 as a function of adolescent poly-victimization, controlling for each measure of early-life vulnerability separately and then controlling for all three simultaneously. Our results indicated that adolescent poly-victimization continued to predict “p” in each of these models (see Table 2). Together, the results in Tables 1 and 2 suggest a cyclical relationship between victimization and psychopathology, wherein children with early-life emotional/behavioral problems and greater family history of mental disorder are at higher risk of being victimized in adolescence, and children victimized in adolescence are at higher risk of developing additional psychiatric symptoms by the time they reach age 18. Importantly, these results also indicate that the association between adolescent poly-victimization and early-adult psychopathology cannot be solely explained by greater preexisting vulnerability to adult disorder among victimized adolescents.

**Table 2.** Associations Between Adolescent Victimization and Early-Adult Psychopathology Controlling for Preexisting Psychiatric Vulnerabilities

Predictors (*z*-scored)				Early-adult psychopathology (age 18) (“p”; *M* = 100, *SD* = 15)				
				(1)	(2)	(3)	(4)	(5)
Adolescent victimization (ages 12-18)				7.09** [6.51, 7.66]	6.63** [6.02, 7.24]	6.90** [6.33, 7.47]	6.91** [6.33, 7.49]	6.46** [5.85, 7.07]
Mental health problems (age 12)				—	2.37** [1.78, 2.97]	—	—	2.00** [1.39, 2.62]
Emotional and behavioral problems (age 5)				—	—	1.72** [1.13, 2.31]	—	0.92* [0.27, 1.57]
Proportion of family members with any disorder				—	—	—	2.02** [1.37, 2.67]	1.48** [0.81, 2.15]

Note: In all linear mixed models, the three preexisting psychiatric vulnerabilities and adolescent poly-victimization were all standardized to a *z*-score with mean of 0 and a standard deviation of 1 to facilitate comparison across measures, whereas “p” remains scaled to a mean of 100 with a standard deviation of 15. 95% confidence intervals are reported in brackets.

* *p* < .01. ** *p* < .001.

#### Is the relationship between adolescent victimization and “p” accounted for by childhood victimization or do victimization in adolescence and victimization in childhood each contribute uniquely to “p”?

Another possibility, suggested by research on “sensitive period effects” (Andersen et al., 2008; Dunn, McLaughlin, Slopen, Rosand, & Smoller, 2013; Kaplow & Widom, 2007), is that victimization in early life increases both a child’s risk of revictimization as well as his or her risk of psychopathology. Consequently, the association between adolescent victimization and adult mental health may arise simply because victimized children are at increased risk of both revictimization in adolescence (Finkelhor, Ormrod, & Turner, 2007b) and psychiatric disorders in adulthood. Like the previous two models, this model also posits a noncausal relationship between adolescent exposure and “p,” suggesting instead that most early-adult psychopathology is attributable to victimization in childhood.

Alternatively, both childhood victimization and adolescent victimization could make independent contributions to early-adult mental health, consistent with research indicating a dose-response relationship between accumulation of adverse life experiences and risk of psychiatric illness (e.g., Anda et al., 2006; Anda et al., 2002). This model suggests that victimization exposure exerts a deleterious effect on early-adult mental health regardless of whether it occurs before or after the transition into adolescence.

We tested these two possibilities using a linear mixed model, predicting “p” at age 18 as a function of adolescent poly-victimization, controlling for poly-victimization in childhood. Our model indicated that both poly-victimization in childhood (*b* = 1.68, *p* < .001) and poly-victimization in adolescence (*b* = 6.78, *p* < .001) made unique contributions to the prediction of “p,” suggesting that E-Risk members with higher levels of victimization exposure during each time period tended to score higher on “p” than E-Risk members with less exposure (see Table S8 in the Supplemental Material). This result suggests that both childhood victimization and adolescent victimization exert independent effects on young-adult mental health, consistent with existing literature indicating that the best predictor of adult psychopathology is an individual’s cumulative exposure.

### Is the association between victimization and psychopathology wholly accounted for by shared genetic and environmental influences?

Our inclusion of statistical controls for childhood victimization and psychiatric vulnerability allowed us to rule out two plausible “third variables” that might explain the association between adolescent victimization and young-adult psychopathology. However, the association could be attributable to other factors shared by children growing up in the same family, including socioeconomic, neighborhood, or cultural conditions. In addition, a second prominent challenge to interpreting the association between victimization and psychopathology is that both are under genetic influence. For example, in E-Risk, MZ twin pairs are more highly correlated in their “p factor” scores than are DZ twins (*r*s = .51 vs. .26). This is expected, given the well-known heritability of most psychiatric disorders (Polderman et al., 2015). More surprising is that MZ twin pairs are also more highly correlated in their victimization experiences than are DZ twins (*r*s = .50 vs. .32). This suggests the presence of genetic effects on environmental exposures, a gene-environment correlation (G-E; see Table S9 in the Supplemental Material, which provides the within-twin-pair correlation coefficients for measures of adolescent poly-victimization and psychopathology).

We used the twin design of the E-Risk Study to account for shared environmental and genetic confounding effects on the association between victimization and psychopathology. Specifically, a test of the association among twins reared together examines whether victimization and psychopathology covary solely because of environmental factors shared by the siblings. A test of this association limited to MZ twins reared together, who share 100% of their genes in common, can go one step further and also examines whether victimization and psychopathology covary because of shared genetic propensity. Figure S3 in the Supplemental Material shows the extent of phenotypic discordance as a function of victimization discordance in the E-Risk cohort.

We parsed the effect of adolescent poly-victimization on “p” into *between-twin pair effects* and *within-twin pair effects* using a linear regression model with the following specification:

$$E\left(Y_{ij}\right)=\beta_{0}+\beta_{w}\left(X_{ij}-{\bar{X}}_{i}\right)+\beta_{B}{\bar{X}}_{i},$$

where *i* is used to index twin pairs and *j* represents individual twins within pairs, so *E*(*Y_ij_*) and *X_ij_* represent, respectively, the predicted score on “p” and the adolescent poly-victimization score for the *j*th twin of the *i*th pair, whereas X–i represents the mean adolescent poly-victimization score for both twins within the *i*th pair. The between-twin-pair regression coefficient (β_B_) estimates whether pairs of twins with higher average poly-victimization tend to have higher “p” at age 18 years. In contrast, the within-twin-pair regression coefficient (β_w_) estimates whether the twin with higher poly-victimization than his or her co-twin tends to also have higher “p” than his or her co-twin (Carlin, Gurrin, Sterne, Morley, & Dwyer, 2005).

As shown in Table 3, within-twin-pair differences in victimization among both DZ and MZ twins were significantly associated with differences in “p,” such that the co-twin who experienced more adolescent poly-victimization had a higher “p” at age 18 (*b* = 5.96, *p* < .001). We found a similar pattern when the analysis was repeated using only MZ twins (*b* = 4.95, *p* < .001). These findings indicate that the association between victimization and “p” could not be fully explained by shared family-wide environmental factors or genetic factors, suggesting the possibility of an environmentally mediated pathway from greater victimization exposure in adolescence to more psychiatric symptoms in early adulthood.

**Table 3.** Results From Discordant-Twin Models of Adolescent Poly-Victimization and Early-Adult Psychopathology (“p”)

Effect	All twins(*N*_pairs_ = 1,019)			MZ twins(*N*_pairs_ = 579)		
	β	95% CI	*p*	β	95% CI	*p*
Family-wide (β_B_)	8.98	[8.06, 9.90]	< .001	9.64	[8.35, 10.93]	< .001
Unique (β_W_)	5.97	[4.84, 7.09]	< .001	4.95	[3.36, 6.53]	< .001

Note: Results from two discordant-twin models that predict “p” as a function of both within-twin and between-twin differences in adolescent poly-victimization. Estimates are reported in standardized units where 15 points equals 1 standard deviation. MZ = monozygotic; CI = confidence interval. Family-wide indicates between-twin-pair difference; unique indicates within-twin-pair difference.

Although the twin-difference model effectively rules out the confounding effects of shared environmental influences (and genetic influences, in the case of MZ twins) on the association between victimization and “p,” it does not rule out the possibility that twin-idiosyncratic differences account for the association between victimization and “p.” Thus, we went one step further and added additional covariates to the regression models to account for twin-specific (environmentally mediated) differences in preexisting vulnerabilities to psychiatric problems; specifically, we added two covariates that measured, respectively, within-pair twin differences in childhood emotional and behavioral problems and in a count of mental health problems assessed at age 12. After accounting for these twin-idiosyncratic differences, we continued to observe associations between twin differences in victimization and twin differences in “p,” in the full sample (*b* = 5.62, 95% confidence interval [CI] = [4.43, 6.80], *p* < .001) and, importantly, among MZ twins (*b* = 4.60, 95% CI = [2.92, 6.28], *p* < .001).

We also used bivariate biometric twin modeling to decompose phenotypic variation in adolescent poly-victimization, “p,” and their association into three components: additive genetic (A), shared environmental (C), and nonshared environmental influences (E) (see Fig. S4 in the Supplemental Material). The results of the bivariate model show that 63% (95% CI = [32%, 94%]) of the association between victimization and psychopathology is a function of shared genetic variation (i.e., the same genes influencing both variables), 8% (95% CI = [0%, 36%]) is accounted for by shared environmental factors, and 29% (95% CI = [21%, 37%]) is accounted for by nonshared environmental factors. Taken together, these results indicate that the association between victimization and psychopathology is complex, with the majority of the association accounted for by shared genetic factors, but some that is also attributable to an independent environmentally mediated effect. This finding of a significant contribution of nonshared environmental effects (E) is consistent with the results of our discordant-twin analyses, in that it suggests part of the association between adolescent poly-victimization and “p” is attributable to factors other than shared environmental and genetic risk factors.

#### What about the residual factors from the bi-factor model of “p”?

Whereas the correlated-factors model identifies higher order propensities to distinct forms of psychopathology (e.g., internalizing, externalizing, and thought disorder symptoms and disorders; see Fig. S2a), the hierarchical bi-factor model suggests that there is one common liability to all these forms of psychopathology and also a set of residual factors that influence a smaller subset of symptoms and disorders (see Fig. S2b). However, the meaning and significance of these residual factors has yet to be clarified in the emerging literature about a general factor of psychopathology. Thus far, we have shown that the associations between victimization and each of the three higher order propensities (internalizing, externalizing, and thought disorders) are similar and nonspecific, and this nonspecificity is parsimoniously captured in the association between victimization and the general factor “p.” This leaves the question: Is there any association between victimization and the residual factors from the bi-factor model? Table S10 in the Supplemental Material shows that the associations between victimization and the residual (i.e., independent of “p”) internalizing, externalizing, and thought disorder factors from the bi-factor model specification of psychopathology are 44%, 48%, and 21% the size of the associations between victimization and these higher order factors from the correlated-factors model (which are not independent of “p”). Moreover, in the stringent MZ twin-difference model, we find no significant associations between victimization and the residual internalizing (*b* = 0.26, 95% CI = [–1.73, 2.24], *p* = .799) and thought disorder (*b* = 1.28, 95% CI = [−0.92, 3.48], *p* = .255) factors. We do, however, find a significant association with the residual externalizing factor (*b* = 2.88, 95% CI = [1.48, 4.28], *p* < .001), consistent with research on the relationship between chronic stress and “p” (Snyder, Young, & Hankin, 2017a). This finding suggests that victimization may be related to young adults’ antisocial and substance-use problems independently of their general propensity to psychopathology. Taken together, however, these results are consistent with the hypothesis that “p” accounts for most of the shared variation between victimization and multiple different forms of psychopathology.

## Discussion

The present study makes two contributions to understanding the relationship between victimization exposure and compromised mental health. First, we addressed the issues of exposure equivalence and outcome specificity by showing (a) that all forms of adolescent victimization studied predicted poorer young-adult mental health with similar effect sizes, and (b) that each form elevated general liability to disorder across multiple psychiatric spectra. Second, we used our longitudinal twin design to rule out four of the most plausible, noncausal explanations for the association between victimization and psychopathology, increasing confidence that causal effects are likely present, although not proving causation.

Some readers may reasonably question the necessity of research that aims to test a causal link between victimization exposure and psychopathology, perhaps wondering how their association could be noncausal. In fact, however, the assumption that such experiences necessarily mold the person is not an open-and-shut case. A series of influential public addresses (e.g., Scarr, 1992) and popular science books (Harris, 2009; Pinker, 2003; Rowe, 1995) has suggested that “the nurture assumption” may be exaggerated and deserves to be empirically scrutinized. Taking up the challenge, a companion report to this article from the E-Risk cohort failed to find evidence of a direct, environmentally mediated effect of victimization exposure on cognitive functioning (Danese et al., 2017).

In the domain of mental health, several recent empirical tests have reported that much (if not all) of the association between victimization and psychopathology may be attributable to common shared environmental and/or genetic risk factors (Berenz et al., 2013; Bornovalova et al., 2013; Dinkler et al., 2017; Dinwiddie et al., 2000; Shakoor et al., 2015; Young-Wolff et al., 2011). These findings are partially confirmed by the present study, as our bivariate twin analysis indicated that the majority of the phenotypic correlation was attributable to genetic influences. Thus, the phenotypic covariation of adolescent poly-victimization with young-adult psychopathology seemed to be driven substantially by shared genetic liability. Nevertheless, the present study diverges from these previous reports in finding that the association between victimization and psychopathology was also partly attributable to common, nonshared environmental influences. This finding suggests two possibilities: (a) that part of the covariation is driven by one or more unique environmental “third variables,” or (b) that part of the covariation reflects an environmentally mediated, causal effect of adolescent victimization on adult psychopathology.

In addition to ruling out the possibility that the association between victimization and psychopathology might be wholly attributable to shared genetic or family-wide influences, the present study also leveraged informant-report data and analyses of within-individual change to rule out additional alternatives. First, we used reports from co-twins and parents to rule out the possibility that E-Risk members’ reports of victimization in adolescence were solely driven by psychiatric symptoms at the time of victimization recall (ruling out mono-method bias). Second, longitudinal within-individual analyses showed that victimization predicted worse mental health in early-adulthood controlling for preexisting psychiatric vulnerabilities (ruling out reverse causation). Third, adolescent victimization made unique contributions to worse mental health in early adulthood, apart from childhood victimization (ruling out revictimization).

Together, these findings add to a growing literature suggestive of a causal relationship between victimization exposure and poor mental health. Table S11 in the Supplemental Material lists the Hill Criteria (Hill, 2015), which are used in epidemiology for evaluating causality. The table summarizes the current state of knowledge and the new contributions made by the E-Risk analyses.

Although much of the previous research on the mental health effects of victimization has focused on victimization in childhood (e.g., maltreatment, neglect, sexual abuse), the present study extends this research by directing attention to victimization in adolescence and examining the mental health effects of a wider array of exposures perpetrated by a wider range of actors. Our findings contribute to research on adolescent victimization in two ways. First, we show that adolescent victimization and childhood victimization each make independent contributions to the prediction of early-adult mental health, consistent with “allostatic load” or “cumulative effects” models of mental and physical disease (e.g., Danese & McEwen, 2012). Second, our results suggest that adolescent poly-victimization exerts a relatively stronger effect on early-adult mental health than childhood poly-victimization, as indicated by a significantly larger effect size with no overlap in CIs (*b =* 6.78, 95% CI = [6.20, 7.36] vs. *b =* 1.68, 95% CI = [1.05, 2.30], respectively). The reason for this difference in effect size is unclear. One possibility is that exposures in adolescence are better predictors of early-adult psychopathology because they happened more recently. A second possibility is that our self-report measure of adolescent victimization may more accurately capture victimization exposures than our parent-report measures of victimization in childhood (which may have underdetected these experiences). A third possibility is that our self-report measures of adolescent victimization may have been influenced by contemporaneous psychiatric symptoms, thereby inflating associations to some degree. We have shown, however, that parental and co-twin reports of adolescent victimization also predicted early-adult psychopathology, which argues against this explanation. A final, intriguing possibility is that our results arise because of developmental differences in vulnerability to the negative mental health consequences of adverse events. This explanation would be consistent with both the epidemiological literature, which shows a relative peak in the onset of mental disorder during adolescence (Kessler et al., 2005; Kim-Cohen et al., 2003), and empirical research suggesting that adolescence may function as a “sensitive period” for the development of neural circuitry known to play a role in the generation of psychiatric symptoms (Fuhrmann, Knoll, & Blakemore, 2015; Paus, Keshavan, & Giedd, 2008).

These results contribute to ongoing debate regarding whether or not the psychiatric sequelae of victimization exposure differ as a function of exposure type. Consistent with previous studies demonstrating that poor mental health is similarly influenced by a wide array of different types of adverse exposures (Edwards et al., 2003; Green et al., 2010; Kessler, Davis, & Kendler, 1997; Putnam et al., 2013; Scott et al., 2010; Vachon et al., 2015), we found that severe exposure to each of the seven types of adolescent victimization assessed in our study was associated with significantly higher “p” scores at age 18 years. Thus, our study replicates previous results concerning the negative mental health effects of abuse, neglect, and maltreatment and extends these findings to show that novel, less-studied forms of victimization in the modern world (i.e., Internet/phone victimization) also appear to be harmful. Although the results show that some types of adolescent victimization (i.e., maltreatment, neglect, sexual abuse) were associated with larger increases in “p” than other types of victimization, it appears that these differences are largely attributable to the excess of poly-victimization associated with these exposures.

The finding that each severe exposure predicted increased symptomatology across all three of the correlated factors subsumed by “p” (internalizing, externalizing, and thought disorders) adds additional support to the notion that the negative mental health effects of victimization exposure are generally nonspecific and tend to increase risk of multiple different psychiatric disorders (Keyes et al., 2012; Vachon et al., 2015). It also may help to explain why individuals diagnosed with a psychiatric disorder who have a history of victimization typically endorse greater numbers of symptoms and experience higher psychiatric comorbidity than nonvictimized individuals with the same diagnosis (Agnew-Blais & Danese, 2016; Putnam et al., 2013; Widom et al., 2007). Although we note some heterogeneity in the magnitude of the association between specific exposures and individual factor scores (e.g., severe neglect or sexual victimization seem to predict larger increases in internalizing and thought disorder symptoms relative to externalizing symptoms), the magnitudes of these differences are fairly small relative to the magnitude of the overall effects, suggesting that the psychiatric disturbance attributable to victimization exposure is manifest with little specificity (see Fig. 1).

Findings from this study should be interpreted in light of several limitations. First, our data were collected from a single cohort born in the United Kingdom in the 1990s. Future research is needed to assess whether these results can be generalized to populations born at different times and in different locations. Second, the sample comprised twins, and thus our results may not generalize to singletons. However, the prevalence of psychopathology and victimization does not differ between singletons and twins (Fisher et al., 2015; Gjone & Nøvik, 1995). Third, our data include only individuals reared in a family environment. Exposure to particularly severe or unusual victimization experiences, such as growing up in an institution characterized by profound material and/or social neglect (Zeanah et al., 2009), may lead to different patterns of emotional and behavioral problems from those analyzed here (see Sheridan & McLaughlin, 2014). Fourth, our sample did not contain sufficient numbers of victimized twin-pairs for us to test whether twins discordant for individual types of adolescent victimization exposure differed on “p.” This limitation means that although we demonstrated that each of seven types of adolescent victimization predicted “p” controlling for exposure to the six other victimization types, we cannot comment on the extent to which any individual type of victimization assessed by our study predicts early-adult psychopathology independent of shared family-wide and genetic risk. Fifth, our assessment of psychiatric outcomes was limited to a single assessment wave at age 18. The implications of this design feature for our findings are not clear. On one hand, many young adults who experience psychiatric symptoms following victimization may experience symptom remission as they age, suggesting that our estimates of the effect of adolescent victimization on adult mental health may be biased upward. On the other hand, many victimized individuals may also develop and then recover from mental disorder between the ages of 12 and 18, or develop frank psychiatric symptoms only later in life, in which case our estimates of the effect of adolescent victimization on later mental disorder may be biased downward. Future studies that employ repeated assessments of mental disorder over time can address this issue.

Finally, only observational studies can ethically test the association between victimization and psychopathology; experiments are not possible. Therefore, proving a causal effect of victimization on mental health is methodologically challenging (Jaffee, 2017). The present study has been able to rule out several prominent noncausal explanations for the association, but we cannot emphasize enough that our study does not prove causation. We have ruled out mono-method bias, reverse causation, and confounding by genetic factors and by family-wide environmental factors, and although we cannot rule out all possible confounds due to possible twin-idiosyncratic environmental differences, we were able to also rule out twin-specific differences in preexisting vulnerability to mental health problems through which these twin-idiosyncratic environmental differences would most likely operate. Although total confounding is increasingly a more remote possibility, causation remains unproven.

Despite these limitations, our findings have several implications for clinical practice and public health. First, they suggest that programs aimed at reducing the rates at which adolescents experience victimization may be an effective means of reducing the burden of mental disorder in early adulthood (which, it is hoped, will translate into a lower incidence of mental disorder across the life course). Second, our findings highlight the importance of developing harm-reduction programs designed to help victimized children and adolescents cope with their adverse experiences in a way that minimizes risk of subsequent psychopathology. These interventions may be particularly beneficial for adolescents exposed to multiple forms of victimization, as these individuals develop the widest array of psychiatric symptoms by early adulthood. Importantly, such interventions are likely to be effective even if victimization exposures are merely epiphenomena that do little more than “tag” individuals at high risk for subsequent psychopathology from other causes.

Third, the relatively homogeneous effects of severe exposure to each type of victimization ascertained in our study suggest that clinicians may wish to ask psychiatric patients about past exposure to multiple different types of victimization, rather than limiting their assessment to only common, physical exposures such as abuse or maltreatment. Similarly, the broad and relatively nonspecific associations between victimization and mental health suggest that interventions aimed at minimizing victimization exposure—or reversing any deleterious changes in neurobiology and behavior following victimization exposure—may have equally broad and comprehensive benefits.

Finally, in addition to supporting the development of targeted interventions for at-risk adolescents, our results also encourage research aimed at understanding the proximal processes through which victimization might exert psychopathological effects. Because even very different types of victimization appear to predict similarly poor mental health, finding biomarkers (e.g., alterations in brain activity, cognitive task performance, hypothalamic-pituitary-adrenal axis hormones, or immune biomarkers) specific to an individual type of victimization will likely be challenging. Consequently, our research suggests that future transdiagnostic studies should focus on understanding the biological and psychological sequelae common to many forms of victimization exposure. We hope that continued progress in this area will set the stage for a substantial reduction in psychiatric morbidity.

## Supplementary Material

Supplementary material
