# Supplementary material for: Adolescent Victimization and Early-Adult Psychopathology: Approaching Causal Inference Using a Longitudinal Twin Study to Rule Out Noncausal Explanations
Source: Clin Psychol Sci. 2017 Dec 12;6(3):352–71. doi: 10.1177/2167702617741381 (PMC5952301; doi:10.1177/2167702617741381)
Supplement: Supplementary material [file SCHAEFER_SUPPLEMENTAL_V9_20170927_clean.pdf]

SUPPLEMENTARY MATERIALS TO SCHAEFER ET AL.,

ADOLESCENT VICTIMIZATION AND EARLY-ADULT PSYCHOPATHOLOGY:

APPROACHING CAUSAL INFERENCE USING A LONGITUDINAL TWIN STUDY

TO RULE OUT NON-CAUSAL EXPLANATIONS

The design of the E-Risk Study is captured in **Supplemental Figure 1**. These Supplementary Materials contain additional details about the measurement of victimization experiences in childhood and adolescence, and about the measurement of early-adult psychopathology at age 18. In addition, they contain supplemental figures and tables to accompany statistical analyses reported in the Main Article.

Assessment of victimization

Assessment of victimization in childhood.

We have previously reported evidence on the reliability and validity of our measurement of childhood victimization (Danese et al., 2016). Here we summarize the method.

A team of interviewers visited each family at home when the twins reached ages 5, 7, 10 and 12 years. Each home-visit interview was guided by a series of questions in a booklet. Based on these interviews with the mothers, each interviewer coded in the booklet her initial impression of whether or not she thought a child had been maltreated. The interviewers also recorded notes about their experiences in the home, and if an interviewer was worried about a child, she met with the fieldwork coordinator to debrief. (Sometimes, the Study had to make a referral to help a child.) Codes, notes, and the fieldwork coordinator's narratives from the debriefs have been saved over the years to create a dossier for each child with cumulative information about exposure to domestic violence between the mother and her partner; frequent bullying by peers; physical maltreatment by an adult; sexual abuse; emotional abuse and neglect; and physical neglect. All the component measures are outlined briefly below.

Physical domestic violence. Mothers reported about perpetration by and victimization of 12 forms of physical violence (e.g., slapping, hitting, kicking, strangling) from the Conflict Tactics Scale (CTS) (Straus & Gelles, 1990), on three assessment occasions during the child's first decade of life (when the children were 5, 7, and 10 years of age). Reports of either perpetration or victimization constituted evidence of physical domestic violence. The CTS has between-partner inter-rater reliabilities of 0.76 for perpetration and 0.82 for victimization

(Magdol, Moffitt, Caspi, & Silva, 1998). Families in which no physical violence took place were coded as 0 (55.2%); families in which physical violence took place on one occasion were coded as 1 (28.0%); and families in which physical violence took place on multiple occasions were coded as 2 (16.8%).

Bullying by peers. Experiences of victimization by bullies were assessed using both mothers' and children's reports. During the interview, the following standard definition of bullying was read out: "Someone is being bullied when another child (a) says mean and hurtful things, makes fun, or calls a person mean and hurtful names; (b) completely ignores or excludes someone from their group of friends or leaves them out on purpose; (c) hits, kicks, or shoves a person, or locks them in a room; (d) tells lies or spreads rumors about them; and (e) other hurtful things like these. We call it bullying when these things happen often, and when it is difficult to make it stop. We do not call it bullying when it is done in a friendly or playful way." Mothers were interviewed when children were 7, 10, and 12 years old and asked whether either twin had been bullied by another child, responding never, yes, or frequently. We combined mothers' reports at child age 7 and 10 to derive a measure of victimization during primary school. Mothers' reports when the children were 12 years old indexed victimization during secondary school. During private interviews with the children when they were 12 years old, the children indicated whether they had been bullied by another child during primary or secondary school. When a mother or a child reported victimization, the interviewer asked them to describe what happened. Notes taken by the interviewers were later checked by an independent rater to verify that the events reported could be classified as instances of bullying operationally defined as evidence of (a) repeated harmful actions, (b) between children, and (c) where there is a power

differential between the bully and the victim. Although inter-rater reliability between mothers and children was only modest (kappa = 0.20–0.29), reports of victimization from both informants were similarly associated with children's emotional and behavioral problems, suggesting that each informant provides a unique but meaningful perspective on bullying involvement (Shakoor et al., 2011). We thus combined mother and child reports of victimization to capture all instances of bullying victimization for primary and secondary school separately: reported as not victimized by both mother and child; reported by either mother or child as being occasionally victimized; and reported as being occasionally victimized by both informants or as frequently victimized by either mother or child or both (Bowes et al., 2013). We then combined these primary and secondary school ratings to create a bullying victimization variable for the entire childhood period (5–12 years). Children who were never bullied in primary or secondary school or occasionally bullied during one of these time periods were coded as 0 (55.5%); children who were occasionally bullied during primary and secondary school, or frequently bullied during one of these time periods were coded as 1 (35.6%); and children who were frequently bullied at both primary and secondary school were coded as 2 (8.9%).

Physical and sexual harm by an adult. When the twins were aged 5, 7, 10 and 12, their mothers were interviewed about each twins' experience of intentional harm by an adult. At age 5 we used the standardized clinical protocol from the MultiSite Child Development Project (Dodge et al., 1990; Lansford et al., 2002). At ages 7, 10, and 12 this interview was modified to expand its coverage of contexts for child harm. Interviews were designed to enhance mothers' comfort with reporting valid child maltreatment information, while also meeting researchers' responsibilities for referral under the U.K. Children Act. Specifically, mothers were asked

whether either of their twins had been intentionally harmed (physically or sexually) by an adult or had contact with welfare agencies. If caregivers endorsed a question, research workers made extensive notes on what had happened, and indicated whether physical and/or psychological harm had occurred. Under the U.K. Children Act, our responsibility was to secure intervention if maltreatment was current and ongoing. Such intervention on behalf of E-Risk families was carried out with parental cooperation in all but one case. No families left the study following intervention. Over the years of data collection, the study developed a cumulative profile for each child, comprising the caregiver reports, recorded debriefings with research workers who had coded any indication of maltreatment at any of the successive home visits, recorded narratives of the successive caregiver interviews, and information from clinicians whenever the Study team made a child-protection referral. The profiles were reviewed at the end of the age–12 phase by two clinical psychologists. Inter-rater agreement between the coders was 90% for cases for whom maltreatment was identified (100% for cases of sexual abuse), and discrepantly coded cases were resolved by consensus review. These were coded as: 0 = no physical harm at any age; 1 = probable physical harm at any age; and 2 = definite physical harm at any age. There were 15.0% of children coded as probably being exposed to physical harm and 5.1% as definitely physically harmed by 12 years of age. There were 1.5% of the children coded as being exposed to sexual abuse.

Emotional abuse and neglect were coded from research workers' narratives of the home visits at ages 5, 7, 10, and 12. We coded quite severe examples of parental behavior observed. For example, a mother who had schizophrenia screamed and swore at the children throughout the home visit. As another example, a father who was drunk during the home visit repeatedly spoke

abusively to the children in front of the research workers. We found that coders could not empirically separate emotional abuse and emotional neglect in a reliable way and thus such experiences were coded together as emotional abuse/neglect. Inter-rater agreement between the coders exceeded 85% for cases with emotional abuse and neglect, and discrepant cases were resolved by consensus review. Children with no evidence of emotional abuse/neglect were coded as 0 (88.3%), those where there was some indication of emotionally inappropriate/potentially abusive or neglectful behavior were coded as 1 (8.7%), and where there was evidence of severe emotional abuse/neglect the children were coded as 2 (3.0%).

Physical neglect. The cumulative observations of the physical state of the home environment documented by the research workers during home visits to the twins at ages 5, 7, 10 and 12 were reviewed by two raters for evidence of physical neglect. This was defined as any sign that the caretaker was not providing a safe, sanitary, or healthy environment for the child. This included the child not having proper clothing or food, as well as grossly unsanitary home environments. (However, this did not include a family living in a deprived or crime-ridden neighborhood.) Inter-rater agreement between the coders was 85%, and discrepantly coded cases were resolved by consensus review. Children with no evidence of physical neglect were coded as 0 (90.9%), those for whom there was an indication of minor physical neglect were coded as 1 (7.1%), and where there was evidence of severe physical neglect the children were coded as 2 (2.0%).

Assessment of victimization in adolescence.

We have previously reported evidence on the reliability and validity of our measurement of adolescent victimization (Fisher et al., 2015). Here we summarize the method.

Within each pair of twins in our cohort, co-twins were interviewed separately at age 18 by a different research worker and were assured of the confidentiality of their responses. The participants were advised that confidentiality would only be broken if they told the research worker that they were in immediate danger of being hurt, and in such situations the project leader would be informed and would contact the participant to discuss a plan for safety.

Our adapted version of the JVQ comprised 5 questions asking about maltreatment, 5 about neglect, 7 about sexual victimization, 6 about family violence, 10 about peer/sibling victimization, 3 about internet/mobile phone victimization, and 9 about crime victimization. Each JVQ question was asked for the period 'since you were 12'. Participants were given the option to say "yes" or "no" as to whether each type of victimization had occurred in the reporting period. Research workers could rate each item "maybe" if the participant seemed unsure or hesitant in their response or they were not convinced that the participant understood the question or was paying attention. Items rated as "maybe" were recoded as "no" or "yes" by the rating team based on the notes provided by the research workers. When insufficient notes were available, these responses were recoded conservatively as a "no". Consistent with the JVQ manual (Finkelhor et al., 2011; Hamby et al., 2004), participants were coded as 1 if they reported any experience within each type of victimization category, or 0 if none of the experiences within the category were endorsed. If an experience was endorsed within a victimization category, follow-up questions were asked concerning how old the participant was when it (first) happened, whether the participant was physically injured in the event, whether the participant was upset or distressed by the event; and how long it went on for (by marking the number of years on a Life History Calendar; Caspi et al., 1996). In addition, the interviewer wrote detailed notes based on

the participant's description of the worst event. If multiple experiences were endorsed within a victimization category, the participant was asked to identify and report about their worst experience.

All information from the JVQ interview was compiled into victimization dossiers. Using these dossiers, each of the seven victimization categories was rated by an expert in victimology and 3 other members of the E-Risk team who were trained on using the rating criteria. Ratings were made using a 6-point scale: 0 = not exposed, then 1-5 for increasing levels of severity. The anchor points for these ratings were adapted from the coding system used for the Childhood Experience of Care and Abuse interview (CECA; Bifulco et al., 1994a; Bifulco et al., 1994n), which has good inter-rater reliability (Bifulco et al., 1994a; Bifulco et al., 1997). The CECA is a comprehensive semi-structured interview whose standardized coding system attempts to improve the objectivity of ratings by basing them on the coder's perspective (rather than relying on the participant's judgment) and focusing on concrete descriptions rather than perceptions or emotional responses to the questions, together with considering the context in which the adverse experience occurred.

In our adapted coding scheme, the anchor points of the scale differ for each victimization category, with some focused more on the severity of physical injury that is likely to have been incurred during victimization exposure (crime victimization, family violence, maltreatment), while others are more focused on the frequency of occurrence of victimization (peer/sibling victimization and internet/mobile phone victimization), the physical intrusiveness of the event (sexual victimization), or the pervasiveness of the effects of victimization (neglect). This reflects the different ways in which severity has previously been defined for different types of

victimization (Barnett et al., 1993; Bifulco et al., 1994a). (Given that our sample comprises twins, we also coded if any of the victimization events experienced by each twin had been perpetrated by their co-twin, as it is possible that growing up with a genetically related, same-age child could increase or decrease sibling victimization rates.) Each twin's dossier was evaluated separately and we did not use information provided in the co-twin's dossier about their own or shared victimization experiences to rate direct or witnessed violence exposure for the target twin. High levels of inter-rater reliability were achieved for the severity ratings for all forms of victimization: crime victimization (*intra-class correlation coefficient [ICC]* = 0.89, $p < 0.001$), peer/sibling victimization (*ICC* = 0.91, $p < 0.001$), internet/mobile phone victimization (*ICC* = 0.90, $p < 0.001$), sexual victimization (*ICC* = 0.87, $p < 0.001$), family violence (*ICC* = 0.93, $p < 0.001$), maltreatment (*ICC* = 0.90, $p < 0.001$), and neglect (*ICC* = 0.74, $p < 0.001$).

The ratings for each type of victimization were then grouped into three classes: 0 – no exposure (score of 0), 1 – some exposure (score of 1, 2 or 3), and 2 – severe exposure (score of 4 or 5) due to small numbers for some of the rating points. Combining ratings of 4 and 5 is also consistent with previous studies using the CECA, which have collapsed comparable scale values to indicate presence of "severe" abuse (e.g., Bifulco et al., 1994; Bifulco et al., 1997; Bifulco et al., 1998; Fisher et al., 2011).

The structure of psychopathology at age 18

Using confirmatory factor analysis (CFA), we tested two standard models (Brunner et al., 2012; Rindskopf & Rose, 1988) that are frequently used to examine hierarchically structured constructs: a Correlated-Factors Model with 3 factors (representing Internalizing, Externalizing, and Thought Disorders; **Supplemental Figure 2A**), and a Bi-Factor Model specifying a general

psychopathology factor (labeled "p", **Supplemental Figure 2B**). These models included the 11 observed variables listed in **Supplemental Table 1** (i.e. alcohol dependence, cannabis dependence, tobacco dependence, conduct disorder, ADHD, anxiety, depression, eating disorders, PTSD, psychotic-like experiences, prodromal symptoms).

In CFA, latent continuous factors are hypothesized to account for the pattern of covariance among observed variables. Our confirmatory factor analyses were run as two-level clustered models to account for the nesting of twins within families. Because symptom-level data are ordinal and have highly skewed distributions, we used polychoric correlations when testing our models. Polychoric correlations provide estimates of the Pearson correlation by mapping thresholds to underlying normally distributed continuous latent variables that are assumed to give rise to the observed ordinal variables. As expected, all disorder/symptom scales were positively correlated, with correlations ranging from 0.10 to 0.66 (**Supplemental Table 2**).

All CFA analyses were performed in MPlus v7.4 (Muthen & Muthen, 1998-2013) using the robust maximum likelihood estimator (MLR). The MLR estimator uses a sandwich estimator to provide standard errors that are robust to non-normality and non-independence of observations. We assessed the relative fit of each model in **Supplemental Figure 2** using the Akaike Information Criterion (AIC), Bayesian Information Criterion (BIC) and the Sample Adjusted BIC. We followed the steps outlined at: https://www.statmodel.com/chidiff.shtml to calculate chi-square difference tests for models using the MLR estimator in MPlus.

Do symptoms of mental disorders form three dimensions?

Our first model, a Correlated-Factors Model (see **Supplemental Figure 2A**), has been consistently used in prior research about the structure of psychopathology. This model tests the hypothesis that there are latent trait factors, each of which influences a subset of the measured

diagnoses or symptoms. In our case, we tested three factors representing Externalizing, Internalizing, and Thought Disorders. The model assumes that the Externalizing, Internalizing and Thought Disorder factors may be correlated. We were guided in decisions regarding which disorders loaded on which factors by the Hierarchical Taxonomy of Psychopathology (HiTOP) consortium (https://medicine.stonybrookmedicine.edu/HITOP/AboutHiTOP; Kotov et al., 2017). As such, symptoms corresponding to disorders of substance use (i.e., Alcohol, Marijuana, Smoking) and oppositional behavior (i.e., Conduct Disorder and ADHD) loaded on the Externalizing factor; symptoms corresponding to disorders of distress (i.e., MDE, GAD and PTSD) and eating pathology (i.e., Eating Disorder) loaded on the Internalizing factor; and symptoms corresponding to disorders associated with psychosis loaded on the Thought Disorders factor.

Supplemental Table 3 shows this model with standardized factor loadings and the correlations between the three factors. The model fit statistics were as follows: AIC=42987.116, BIC=43488.486, Sample Adjusted BIC=43205.726. Loadings on each of the three factors were all positive, generally high (all p's < .001) and averaged 0.680 (Externalizing: average loading=0.638; Internalizing: average loading=0.654; Thought Disorders: average loading=0.836). Correlations between the three factors were all positive and ranged from 0.552 between Externalizing and Thought Disorders to 0.756 between Internalizing and Thought Disorders. Thus, this model confirmed that three correlated factors (i.e., Internalizing, Externalizing, and Thought Disorders) explained the structure of the 11 symptom scales examined in the E-Risk twins at age 18.

Is there one general psychopathology factor?

Our second model, the Bi-Factor Model (see **Supplemental Figure 2B**), tested the hypothesis that the symptom measures reflect both general psychopathology and narrower symptom styles of psychopathology. In the Bi-Factor Model, general psychopathology (labeled "p" on **Supplemental Figure 2B**) is represented by a factor that directly influences all of the symptom measures (Lahey et al., 2017). The additional symptom styles are most commonly represented by two factors (i.e., Internalizing and Externalizing), each of which influences a smaller subset of the symptom items (Greene & Eaton, 2017; Laceulle, Vollebergh, & Ormel, 2015; Lahey et al., 2012, 2015; Martel et al., 2016; Murray, Eisner, & Ribeaud, 2016; Olino, Dougherty, Bufferd, Carlson, & Klein, 2014; Patalay et al., 2015; Snyder, Young, & Hankin, 2017). (Note that, to date, most studies of the general factor of psychopathology have not assessed symptoms and disorders related to the Thought Disorder spectrum.) For example, Alcohol Symptoms loaded jointly on the General Psychopathology factor and on the Externalizing style factor, whereas Depression Symptoms loaded jointly on the General Psychopathology factor and on the Internalizing style factor. The style factors represent the constructs of Externalizing and Internalizing over and above General Psychopathology. The classic Bi-Factor Model generally assumes that the specific factors are also uncorrelated (Yung et al., 1999), and we specified our model as such.

We also tested an alternative specification of the Bi-Factor Model that included three symptom-style factors (i.e., Internalizing, Externalizing, and Thought Disorders) in addition to "p". Although the factors derived from this specification correlated with the factors from the more common Bi-Factor specification (i.e., with only Internalizing and Externalizing symptom style factors) at $r > 0.99$, this model fit our data significantly better than the more common

specification (cd = 0.77, χ^2 diff = 30.21, df = 2, p < 0.001). Thus, we retained the three symptom-style factor model for use in all future analyses.

Supplemental Table 3 shows this Bi-Factor Model with standardized factor loadings. Fit statistics were as follows: AIC=42897.350, BIC=43443.787, Sample Adjusted BIC=43135.609 (**Supplemental Table 3**). Loadings on the general factor ("p") were all positive, generally high (all p's < .001) and averaged 0.519; the highest standardized loadings were for psychotic symptoms (0.759 and 0.592), MDE (0.718), eating disorders (0.574), and GAD (0.567). Similarly, the loadings for the three style factors were all positive and averaged 0.507 for Externalizing, 0.270 for Internalizing, and 0.496 for Thought Disorder. Because the Correlated-Factors Model and the Bi-Factor Model are not nested, we could not directly compare them, but AIC and BIC were slightly lower for the Bi-Factor Model.

Additional specification

In addition to the Correlated-Factors and Bi-Factor Models described above, we also considered a Higher-Order Factor Model that specifies "p" as a second-order factor arising from the Internalizing, Externalizing, and Thought Disorder first-order factors (**Supplemental Figure 2C)**. **Supplemental Table 3** shows this model with standardized factor loadings. The model fit statistics were as follows: AIC=42988.345, BIC=43489.715, Sample Adjusted BIC=43206.954 Loadings on each of the three factors were all positive, generally high (all p's < .001) and averaged 0.681 (Externalizing: average loading=0.638; Internalizing: average loading=0.656; Thought Disorders: average loading=0.842). Similarly, first-order factor loadings on "p" averaged 0.782 (Externalizing=0.648; Internalizing=0.858; Thought Disorders=0.841). However, this model fit the data significantly worse than the bi-factor specification (cd = 1.02, χ^2 diff = 105.26, df = 6, p < 0.001). The correlation between "p" derived from the Bi-Factor Model and

from the Higher-Order Factor Model is 0.98; results using the Higher-Order Factor Model "p-factor" are similar to those reported in the Main Article and are available from the authors. Thus, we present results in the main text using only the Bi-Factor and Correlated-Factors Models.

How are disorder-liability factor scores correlated across models?

We output factor scores from the Correlated-Factors Model (A) and the Bi-Factor Model (B), saved them, and calculated their correlations with each other. All three factors from the Correlated-Factors Model were highly correlated with General Psychopathology (r's range from 0.79 for Externalizing to 0.97 for Internalizing), suggesting that, to some extent, all three factors in the Correlated-Factors Model reflected General Psychopathology (**Supplemental Table 4**).

Figure S1. Schedule of victimization and psychopathology assessments in the Environmental Risk (E-Risk) Study.

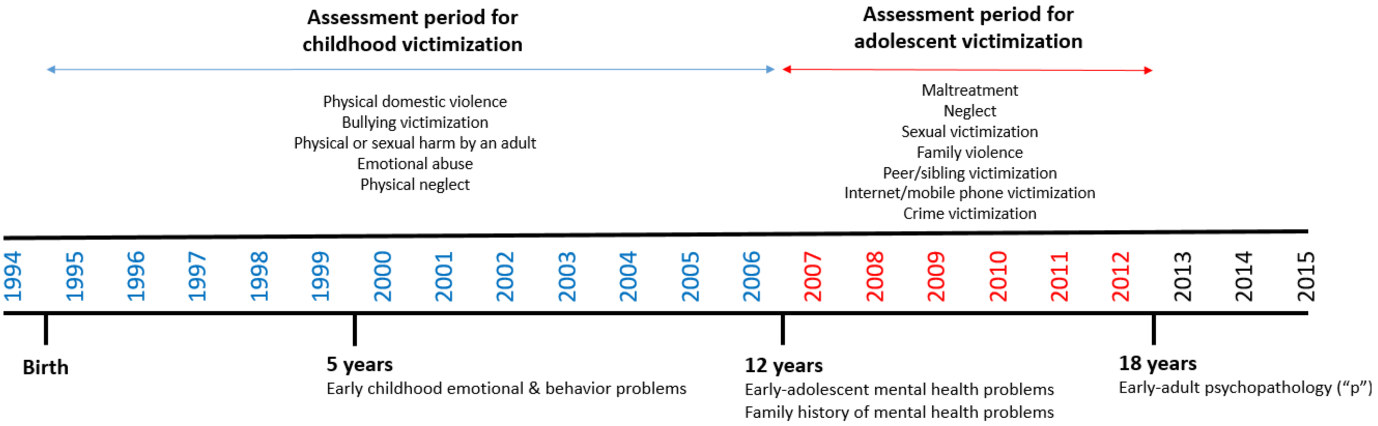

Table S1. Assessment of symptoms of mental disorders in the E-Risk cohort at age 18 years.

Symptom Scale	*N*	*Mean*	*SD*	*Range*	*Example Symptoms*
Alcohol Dependence	2063	1.12	1.66	0 - 9	Tolerance<br>Withdrawal symptoms<br>Unable to cut down<br>Role interference
Cannabis Dependence	2066	0.31	1.24	0 - 8	Tolerance<br>Withdrawal symptoms<br>Unable to cut down<br>Role interference
Tobacco Dependence	2062	0.66	1.59	0 - 7	# of cigarettes per day<br>Hard to refrain from smoking<br>Hate to give up morning cigarette<br>Smoke when sick
Conduct Disorder	2053	2.10	2.22	0 - 9	Fighting<br>Steals (with and without confrontation)<br>Lies, deceives<br>Destroys property
ADHD	2061	5.79	4.29	0 - 18	Can't concentrate, mind wanders<br>Disorganized<br>Fidgety, squirmy, very restless<br>Always on the go, in a hurry, as if driven by a motor
Major Depression	2063	1.81	2.97	0 - 9	Depressed mood<br>Sleep change<br>Fatigue<br>Weight or appetite change

Generalized Anxiety Disorder	2066	1.13	2.15	0 - 7	Restless or keyed up<br>Multiple worries<br>Muscle tension<br>Easily tired
Disordered Eating	2064	0.45	0.88	0 - 5	Lost control over amount eaten<br>Food dominates life<br>Became sick due to eating to uncomfortably full<br>Believe you're fat when others say you're too thin
Post-traumatic stress disorder	2064	0.74	1.74	0 - 8	Flashbacks<br>Hypervigilence<br>Sleep disturbences<br>Avoidance
Psychotic-Like Experiences	2063	0.04	0.27	0 - 3	People have read my thoughts<br>Watched, followed, or spied on<br>Heard voices<br>Under control of some special power
Prodromal Symptoms	2062	0.52	1.31	0 - 8	Thinking is unusual or frightening<br>People or places I know seem different<br>Can't trust anyone<br>Has special abilities or powers

Notes. We assessed the following 11 disorder/symptoms: alcohol dependence, cannabis dependence, tobacco dependence, conduct disorder, attention-deficit hyperactivity disorder (ADHD), major depression, generalized anxiety disorder, post-traumatic stress disorder, disordered eating, psychotic-like experiences, prodromal symptoms. N = # of Study members assessed for each condition; Mean, SD, and range all refer to the number of symptoms endorsed by the full cohort.

Figure S2. Three alternative correlational structures of early-adult psychopathology.

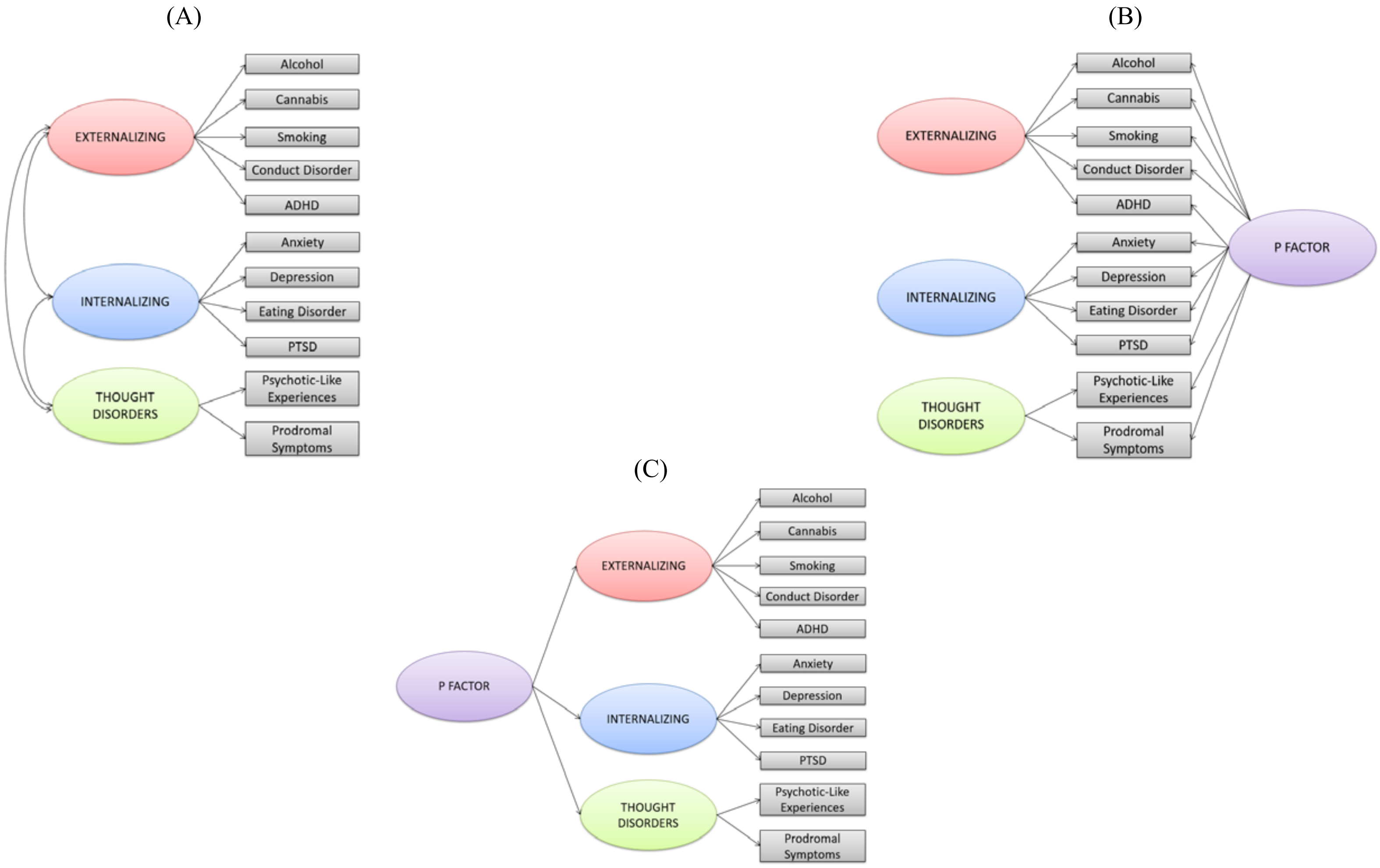

Notes. (A) Correlated-Factors Model, (B) Bi-Factor Model, (C) Higher-Order-Factor Model. Colored ovals represent latent (unobserved) continuous symptom trait factors; grey boxes represent age-18 observed scores on symptom scales corresponding to each disorder. ADHD = attention-deficit hyperactivity disorder, PTSD = post-traumatic stress disorder.

Table S2. Polychoric correlations between psychiatric disorder symptom scales in the E-risk cohort.

	ALC	CANN	SMK	CD	ADHD	GAD	MDE	EAT	PTSD	PSYCH
CANN	0.285									
SMK	0.271	0.639								
CD	0.421	0.628	0.425							
ADHD	0.314	0.344	0.312	0.444						
GAD	0.145	0.143	0.103	0.168	0.313					
MDE	0.298	0.321	0.294	0.298	0.391	0.586				
EAT	0.239	0.210	0.258	0.252	0.346	0.381	0.468			
PTSD	0.167	0.180	0.243	0.192	0.230	0.334	0.480	0.311		
PSYCH	0.131	0.240	0.180	0.295	0.298	0.413	0.413	0.382	0.347	
PDS	0.156	0.419	0.283	0.368	0.401	0.497	0.543	0.416	0.664	0.467

Notes. Higher correlations between some disorders (but not others) support the construction of latent "factor" scores representing the externalizing, internalizing, and thought disorder spectra, whereas the positive correlations between all symptom scales supports the construction of a higher-order factor of liability to general psychopathology (which we label "p"). ALC = alcohol dependence; CANN = cannabis dependence; SMK = tobacco dependence; CD = conduct disorder; ADHD = attention-deficit hyperactivity disorder; MDE = major depressive episode; GAD = generalized anxiety disorder; EAT = eating disorder; PTSD = post-traumatic stress disorder; PSYCH = psychotic-like experiences; PDS = prodromal symptoms.

Table S3. Model fit indices and standardized factor loadings for the Correlated-Factors, Bi-Factor, and Higher-Order-Factor models of early-adult psychopathology.

	Correlated-Factors	Bi-Factor	Higher-Order-Factor
N	2066	2066	2066
# Free Parameters	89	97	89
LL	-21404.558	-21351.675	-21405.172
Scaling factor (c)	1.049	1.057	1.060
AIC	42987.116	42897.350	42988.345
BIC	43488.486	43443.787	43489.715
Sample Adjusted BIC	43205.726	43135.609	43206.954

	Correlated-Factors			Bi-Factor				Higher-Order-Factor			
	Extern-alizing	Intern-alizing	Thought Disorders	p	Extern-alizing	Intern-alizing	Thought Disorders	p	Extern-alizing	Intern-alizing	Thought Disorders
ALC	**0.487**			**0.307**	**0.371**				**0.486**		
CANN	**0.776**			**0.386**	**0.715**				**0.776**		
SMK	**0.586**			**0.338**	**0.483**				**0.586**		
CD	**0.733**			**0.409**	**0.667**				**0.734**		
ADHD	**0.608**			**0.539**	**0.301**				**0.607**		
GAD		**0.661**		**0.567**		**0.368**				**0.662**	
MDE		**0.822**		**0.718**		**0.448**				**0.828**	
EAT		**0.571**		**0.574**		0.083				**0.569**	
PTSD		**0.563**		**0.517**		**0.179**				**0.563**	
PSYCH			**0.761**	**0.592**			**0.673**				**0.750**
PDS			**0.911**	**0.759**			**0.319**				**0.934**
Externalizing								**0.648**			
Internalizing								**0.858**			
Thought Disorders								**0.841**			

Notes. [1]The classic Bi-Factor and Higher-Order Factor Models generally assume that the specific factors are uncorrelated; thus, we set model-estimated correlations between factor scores in each model to 0. Traditional SEM model fit indices such as the chi-square, the comparative fit index (CFI), the Tucker-Lewis index (TLI), and the root-mean-square-error-of-approximation (RMSEA) were unavailable in our clustered model because the frequency table for the latent class indicator model was too large. Factor loadings and correlations in **bold** are significant at $p < 0.05$. ALC = alcohol dependence; CANN = cannabis dependence; SMK = tobacco dependence; CD = conduct disorder; ADHD = attention-deficit hyperactivity disorder; GAD = generalized anxiety disorder; MDE = major depressive episode; EAT = eating disorder; PTSD = post-traumatic stress disorder; PSYCH = psychotic-like experiences; PSD = prodromal symptoms.

Table S4. Correlations among extracted factor scores from the Correlated Factors, Bi-Factor, and Higher-Order-Factor Models of early-adult mental health (N = 2066).

	Correlated Factors			Bi-Factor				Higher-Order-Factor			
	Ext	Int	ThD	Ext	Int	ThD	P	Ext	Int	ThD	P
Externalizing: Correlated Factors	--										
Internalizing: Correlated Factors	0.71	--									
Thought Disorders: Correlated Factors	0.72	0.91	--								
Externalizing: Bi-Factor	0.80	0.19	0.23	--							
Internalizing: Bi-Factor	0.09	0.60	0.29	-0.24	--						
Thought Disorders: Bi-Factor	0.08	0.16	0.48	-0.10	-0.25	--					
P: Bi-Factor	0.79	0.97	0.94	0.26	0.41	0.24	--				
Externalizing: Higher-Order Factor	1.00	0.71	0.72	0.80	0.09	0.08	0.79	--			
Internalizing: Higher-Order Factor	0.71	1.00	0.90	0.18	0.61	0.15	0.97	0.71	--		
Thought Disorders: Higher-Order Factor	0.71	0.89	1.00	0.23	0.26	0.50	0.93	0.71	0.88	--	
P: Higher-Order Factor	0.81	0.97	0.97	0.32	0.42	0.29	0.98	0.81	0.97	0.96	--

Table S5. Increase in early-adult psychopathology ("p") associated with severe exposure to each adolescent victimization type.

Type of Victimization	*Estimate*	*95% CIs*	*p-value*
Model (a):			
Maltreatment	19.63	16.07, 23.19	<0.001
Neglect	18.93	14.78, 23.08	<0.001
Sexual Victimization	20.85	17.08, 24.61	<0.001
Family Violence	11.46	9.59, 13.32	<0.001
Peer/sibling Victimization	10.59	8.93, 12.24	<0.001
Internet/mobile phone	10.52	8.07, 12.98	<0.001
Crime Victimization	12.27	10.76, 13.78	<0.001
Model (b):			
Maltreatment	7.18	3.57, 10.78	<0.001
Neglect	7.81	3.77, 11.86	<0.001
Sexual Victimization	10.94	7.33, 14.55	<0.001
Family Violence	6.83	5.03, 8.62	<0.001
Peer/sibling Victimization	6.30	4.65, 7.95	<0.001
Internet/mobile phone	4.14	1.77, 6.51	0.001
Crime Victimization	7.60	6.08, 9.12	<0.001

Notes. Model (a) displays results from a series of seven separate linear mixed models predicting "p" as a function of severe exposure to a specific type of adolescent victimization, controlling for clustering within families. Estimates here represent coefficients from this model that capture the average difference in "p" between exposed vs. non-exposed Study members. Model (b) displays results from a single, simultaneous linear mixed models predicting "p" as a function of severe exposure to each type of adolescent victimization, controlling for clustering within families. Estimates here represent coefficients from this model that capture the average difference in "p" between exposed vs. non-exposed Study members, controlling for exposure to each of the other 6 victimization types. Because "p" is scaled to a sample mean of 100 and a standard deviation of 15, we report each estimate in standardized units where 15 points equals one standard deviation. CI = confidence interval.

Table S6. Tests of sex differences in the effect of severe exposure to each adolescent victimization type on early-adult psychopathology ("p").

Type of Victimization	*Estimate*	*95% CI*	*p-value*
Male	1.68	0.20, 3.16	0.026
Maltreatment	19.91	14.84, 24.99	<0.001
Male * Maltreatment	-0.53	-7.64, 6.57	0.883
Male	1.61	0.12, 3.10	0.034
Neglect	20.38	13.95, 26.80	<0.001
Male * Neglect	-2.63	-11.04, 5.79	0.541
Male	1.09	-0.38, 2.56	0.147
Sexual Victimization	21.57	12.39, 30.75	<0.001
Male * Sexual Victimization	-1.14	-11.21, 8.93	0.825
Male	1.65	0.13, 3.18	0.034
Family Violence	11.28	8.56, 13.99	<0.001
Male * Family Violence	0.34	-3.38, 4.07	0.856
Male	1.49	-0.04, 3.01	0.056
Peer/sibling Victimization	11.30	8.76, 13.84	<0.001
Male * Peer/sibling Victimization	-1.38	-4.72, 1.97	0.420
Male	1.17	-0.36, 2.70	0.134
Internet/mobile phone	12.52	7.58, 17.46	<0.001
Male * Internet/mobile phone	-2.91	-8.61, 2.79	0.317
Male	1.82	0.31, 3.32	0.018
Crime Victimization	10.35	8.35, 12.36	<0.001
Male * Crime Victimization	5.15	2.11, 8.20	0.001

Notes. Estimates here represent coefficients from a series of separate linear mixed models that each predict "p" as a function of sex, severe exposure to a specific type of adolescent victimization, and the interaction between severe exposure to a specific type of adolescent victimization and sex. Because "p" is scaled to a sample mean of 100 and a standard deviation of 15, we report each estimate in standardized units where 15 points equals one standard deviation. CI = confidence interval.

Table S7. Associations between self-reported, co-twin-reported, and parent-reported exposure to adolescent victimization and early-adult psychopathology ("p").

Reporting source	*N*	*Estimate*	*95% CI*	*p-value*
Self	2062	7.74	7.12, 8.37	<0.001
Co-twin	1994	5.14	4.09, 6.19	<0.001
Parent	1687	5.64	4.45, 6.82	<0.001

Notes. Estimates here represent beta coefficients from three separate linear mixed models that each predict "p" as a function of adolescent victimization exposure reported by each source, controlling for clustering by family. Because "p" is scaled to a sample mean of 100 and a standard deviation of 15, we report each estimate in standardized units where 15 points equals one standard deviation. CI = confidence interval.

Table S8. Associations between adolescent poly-victimization and early-adult psychopathology (“p”), controlling for poly-victimization in childhood.

Predictors (z-scored)	Early-adult psychopathology (age 18) ("p", M = 100, SD = 15)	
	(1)	(2)
Adolescent victimization (ages 12-18)	7.09***	6.78***
	(6.51, 7.66)	(6.20, 7.36)
Childhood victimization (ages 5-12)	-	1.68***
		(1.05, 2.31)

Notes. Estimates shown here represent coefficients from 2 separate linear mixed models using (1) adolescent poly-victimization, and (2) adolescent poly-victimization controlling for childhood poly-victimization to predict “p” at age 18 years. Each predictor variable was standardized to a mean of 0 and a standard deviation of 1 to facilitate comparison across measures. Because “p” is scaled to a sample mean of 100 and a standard deviation of 15, we report each estimate in standardized units where 15 points equals one standard deviation. 95% confidence intervals are reported in parentheses. PV = poly-victimization.
* $p < 0.05$, ** $p < 0.01$, *** $p < 0.001$.

Table S9. Twin correlations between (a) psychiatric disorder symptoms and (b) adolescent poly-victimization in the E-risk cohort, among all twins, MZ twins, and DZ twins.

	All Twins (N's = 1019 to 1044)	MZ Twins (N's = 579 to 586)	DZ Twins (N's = 440 to 458)
Alcohol Dependence	0.370	0.442	0.268
Cannabis Dependence	0.558	0.741	0.277
Tobacco Dependence	0.687	0.792	0.535
Conduct Disorder	0.506	0.565	0.419
ADHD	0.246	0.356	0.093
Generalized Anxiety Disorder	0.286	0.405	0.111
Depression	0.326	0.472	0.104
Eating Disorder	0.325	0.435	0.112
PTSD	0.356	0.471	0.178
Psychotic-like Experiences	0.324	0.452	0.142
Prodromal Symptoms	0.350	0.427	0.238
P-factor (Bi-Factor Model)	0.408	0.513	0.257
Internalizing (Bi-Factor Model)	0.163	0.251	0.045
Externalizing (Bi-Factor Model)	0.493	0.580	0.368
Thought Disorders (Bi-Factor Model)	0.095	0.014	0.050
Internalizing (Correlated-Factors Model)	0.399	0.504	0.248
Externalizing (Correlated-Factors Model)	0.497	0.587	0.368
Thought Disorders (Correlated-Factors Model)	0.363	0.444	0.249
Adolescent poly-victimization	0.522	0.619	0.389

Notes. The table shows polychoric correlations for all variables, except the psychopathology factors and ADHD, which are reported as Pearson correlations.

Figure S3. Mean differences in early-adult psychopathology ("p") within monozygotic and dizygotic twin pairs discordant for 0, 1, 2, and 3+ types of adolescent victimization exposure.

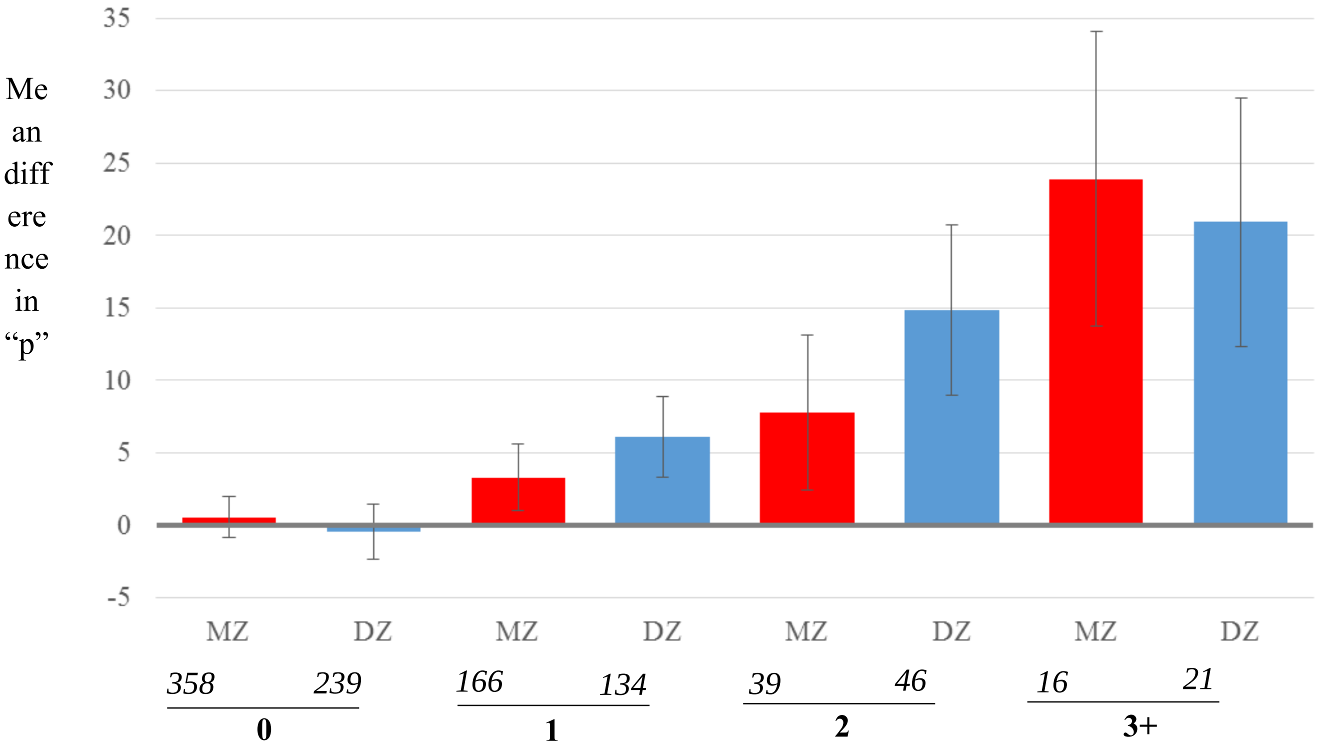

Notes. The numbers beneath the x-axis convey the extent of discordance (bold) as well as the number of twin pairs contributing data to each group mean (italics). For twin pairs with discordance >= 1, we calculated mean difference in "p" as the p-factor score of the twin exposed to greater poly-victimization minus the p-factor score of the twin exposed to less poly-victimization. For twin pairs with discordance = 0, we randomly determined which twin's p-factor score was subtracted from the other. Error bars represent a 95% confidence interval. We scaled "p" to a mean of 100 and a standard deviation of 15. MZ = monozygotic. DZ = dizygotic.

Figure S4. Results from bivariate biometric twin models of adolescent poly-victimization and early-adult psychopathology ("p")

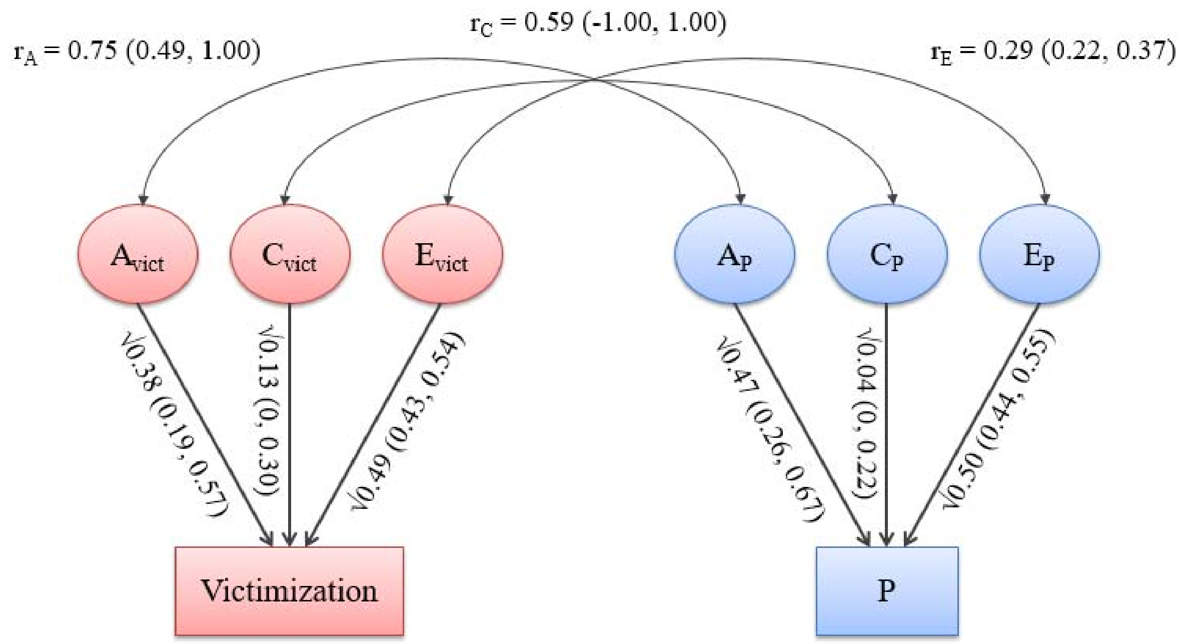

Notes. The bivariate twin model allows for calculation of the extent to which genetic, shared environmental, and non-shared environmental factors contribute to the phenotypic correlation between victimization and psychopathology. Path coefficients represent the proportion of variance in each phenotype that can be attributed to genetic (A), shared environmental (C), and non-shared environmental factors (and measurement error) (E). Correlations r_A, r_C, and r_E represent the genetic, shared environmental, and non-shared environmental correlations between corresponding components of victimization and "p". 95% confidence intervals for each estimate are in parentheses. The proportion of the association accounted for by A, C, and E, can be calculated by multiplying the two path coefficients associated with each type of influence by their respective correlations and dividing by the phenotypic correlation (r_{ph} = 0.50). For example, the proportion of the phenotypic correlation attributable to A is calculated as [$\sqrt{0.38}$ * $\sqrt{0.47}$ * 0.75]/0.50, or 0.63. Expressed as percentages, the proportion of the phenotypic correlation attributable to A, C, and E is 63% (95% CI: 32-94%), 8% (95% CI: 0-36%), and 29% (95% CI: 21-37%), respectively.

Table S10. Associations between poly-victimization with Internalizing, Externalizing, and Thought Disorder factors from the Correlated-Factor Model and with the residual (independent of "p") Internalizing, Externalizing, and Thought Disorder factors from the Bi-Factor Model.

A. Individual-level analysis

	Higher-order dimension from Correlated-Factor Model			Residual factor from Bi-Factor Model of General Psychopathology		
	Est.	95% CI	p-value	Est.	95% CI	p-value
Internalizing	7.74	(7.11, 8.37)	< 0.001	3.40	(2.69, 4.11)	< 0.001
Externalizing	6.53	(5.90, 7.17)	< 0.001	3.11	(2.43, 3.79)	< 0.001
Thought Disorder	7.35	(6.71, 7.99)	< 0.001	1.53	(0.81, 2.25)	< 0.001

B. Twin-level analysis

	Higher-order dimension from Correlated-Factor Model			Residual factor from Bi-Factor Model of General Psychopathology		
	Est.	95% CI	p-value	Est.	95% CI	p-value
Internalizing	4.67	(3.34, 6.00)	< 0.001	1.04	(-0.62, 2.71)	0.219
Externalizing	4.80	(3.60, 5.99)	< 0.001	2.84	(1.60, 4.09)	< 0.001
Thought Disorder	4.94	(3.54, 6.35)	< 0.001	1.00	(-0.38, 2.68)	0.243

Panel A: The associations between victimization and the residual (i.e., independent of "p") Internalizing, Externalizing, and Thought Disorder factors from the Bi-Factor Model specification of psychopathology are 44%, 48%, and 21% the size of the associations between victimization and these higher-order factors from the Correlated-Factors Model (which are not independent of "p"). **Panel B**: In the stringent MZ twin-difference model, we find no significant within-twin associations between victimization and the residual Internalizing and Thought Disorder factors, although there is a significant within-twin association with the residual Externalizing factor, suggesting that victimization may be related to young adults' antisocial and substance-use problems independently of their general propensity to psychopathology. Est. = estimate. CI = confidence interval.

Table S11. Hill Criteria for causation as applied to the relationship between victimization and psychopathology. Hill originally listed 10 criteria, to which we add an 11th: capacity to rule out genetic confounding.

Strength. The larger the association, the more likely that it is causal.	As shown in our study, exposure to each additional type of severe victimization is associated with approximately one-half of a standard deviation increase in "p", an omnibus measure of early-adult psychopathology. This suggests that the magnitude of the average difference in "p" between a Study member who is not victimized in adolescence and a Study member exposed to 3 or more types of severe victimization is akin to the difference in IQ between a person of average intelligence (IQ = 100) and another person with a borderline mental disability (IQ = 77).
Consistency. Consistent findings across different samples in different places and with different characteristics strengthens the likelihood of a causal effect.	Associations between victimization and psychopathology are robust to differences in sample characteristics (age, location, racial/ethnic demographics) (Anda et al., 2006; Green et al., 2010; Scott et al., 2010; Vachon et al., 2015; Widom et al., 2007).
Specificity. The more specific the association is, the more likely that it is causal.	As shown in our study, the relationship between victimization and psychopathology is largely nonspecific. It is not clear how this Hill criterion relates to our finding. There are several explanations for non-specificity. First, the lack of specificity could stem from transdiagnostic mechanisms with very generalized effects. Second, the lack of specificity may be due to the fact that it is difficult to isolate specific effects of individual types of victimization, given the high levels of poly-victimization among victimized individuals. It is likely that our ability to demonstrate specificity will improve alongside our ability to measure and categorize these exposures (see Sheridan & McLaughlin, 2014 for an example of progress in this area).
Temporality. The effect must occur after the cause.	Because children cannot be "assigned" to victimization and because the association between victimization and psychopathology may be reciprocal, the best available methods for establishing the temporality of victimization and psychopathology include using childhood measures of psychopathology or pre-existing psychiatric vulnerabilities as covariates in models that predict future psychopathology as a function of victimization. As shown in our study, adolescent victimization predicts psychopathology net of these controls, consistent with the notion

	that victimization at T1 increases psychopathology at T2.
Biological gradient. Greater exposure should lead to higher incidence of the effect.	As shown in our study, there is clear evidence of a dose-response relationship between victimization and psychopathology, with greater poly-victimization predicting higher “p” in what appears to be a linear fashion (see **Figure 2A**).
Plausibility. A plausible mechanism between cause and effect is helpful.	Several plausible neurobiological and psychological mechanisms have been proposed to explain the effects of victimization on psychopathology (Nemeroff, 2016; Sheridan & McLaughlin, 2014; Teicher, 2002).
Coherence. Concordance between epidemiological and laboratory evidence strengthens the likelihood of a causal effect.	Although “victimization” is difficult to replicate in an experimental context, laboratory studies of animals show that variations in maternal care can affect the development of stress and fear circuitry in offspring (Barr et al., 2004; Liu et al., 1997). Similar alterations have also been seen in children exposed to maltreatment (Harmelen et al., 2013; Herringa et al., 2013). Both sets of findings are consistent with the epidemiological finding of higher rates of psychopathology among victimized individuals.
Experiment. Occasionally it is possible to appeal to experimental evidence.	Because of the ethical and practical dilemmas inherent in “randomizing” individuals to varying levels of victimization, experimental evidence supporting the association between victimization and psychopathology remains weak. However, studies from the Bucharest Early Intervention Project (BEIP), have shown that institutionalized children randomized to early placement in a family caregiving environment (truncating their exposure to profound material and social neglect) show more “normalized” sympathetic nervous system and hypothalamic-pituitary axis activity (McLaughlin et al., 2015) and lower incidence of childhood internalizing symptoms (Humphreys et al., 2015; Zeanah et al., 2009).
Analogy. Evidence of a relationship between a similar cause and effect is helpful.	It is now widely accepted that aspects of both child and brain development are dependent on early experience. Perhaps the most striking demonstration of this principle came from studies of visual development, which demonstrated that depriving animals of normal sight in early life led to enduring alterations in visual perception and visual cortex development (e.g., Wiesel & Hubel, 1963). Just as an abnormal visual environment contributes to long-lasting disruptions in visual processing, exposure to an abnormally deprived or threatening environment during certain sensitive periods is hypothesized to lead to persistent, pathological changes

	in emotion and/or behavior.
Genetic confounding. Ability to rule out the alternative hypothesis of gene-environment correlation.	This was not one of Hill’s criteria. However, emerging understanding of the important role of gene-environment correlations in driving health and development makes it critical to design studies that have the capacity to rule out familial confounds. We leveraged the genetically-informative E-risk Study to show that the association between victimization and psychopathology was not attributable to shared environmental or genetic risk factors, suggesting either (1) that part of the covariation is driven by one or more unique environmental “third variables”, or (2) that part of the covariation reflects an environmentally-mediated, causal effect of adolescent victimization on young-adult psychopathology.

References

Anda, R. F., Felitti, V. J., Bremner, J. D., Walker, J. D., Whitfield, C., Perry, B. D., … Giles, W. H. (2006). The enduring effects of abuse and related adverse experiences in childhood: A convergence of evidence from neurobiology and epidemiology. *European Archives of Psychiatry and Clinical Neuroscience*, *256*(3), 174–186. https://doi.org/http://dx.doi.org/10.1007/s00406-005-0624-4

Barnett, D., Manly, J. T., & Cicchetti, D. (1993). Defining child maltreatment: The interface between policy and research. In D. Cicchetti & S. L. Toth (Eds.), *Child abuse, child development, and social policy* (pp. 7–74). Norwood, NJ: Ablex.

Barr, C. S., Newman, T. K., Shannon, C., Parker, C., Dvoskin, R. L., Becker, M. L., … Higley, J. D. (2004). Rearing condition and rh5-HTTLPR interact to influence limbic-hypothalamic-pituitary-adrenal axis response to stress in infant macaques. *Biological Psychiatry*, *55*(7), 733–738. https://doi.org/10.1016/j.biopsych.2003.12.008

Bifulco, A., Brown, G. W., & Harris, T. O. (1994). Childhood Experience of Care and Abuse (CECA): A retrospective interview measure. *Journal of Child Psychology and Psychiatry, 35*(8), 1419–1435. https://doi.org/10.1111/j.1469-7610.1994.tb01284.x

Bifulco, A., Brown, G. W., Lillie, A., & Jarvis, J. (1997). Memories of childhood neglect and abuse: corroboration in a series of sisters. *Journal of Child Psychology and Psychiatry, 38*(3), 365–374. https://doi.org/10.1111/j.1469-7610.1997.tb01520.x

Bifulco, A., Brown, G. W., Moran, P., Ball, C., & Campbell, C. (1998). Predicting depression in women: the role of past and present vulnerability. *Psychological Medicine*, *28*(1), 39–50.

Bifulco, A., Brown, G. W., Neubauer, A., Moran, P., & Harris, T. (1994). *Childhood Experience of Care and Abuse (CECA) training manual*. London: Royal Holloway College, University of London.

Bowes, L., Maughan, B., Ball, H., Shakoor, S., Ouellet-Morin, I., Caspi, A., … Arseneault, L. (2013). Chronic bullying victimization across school transitions: The role of genetic and environmental influences. *Development and Psychopathology*, *25*(2), 333–46. https://doi.org/http://dx.doi.org/10.1017/S0954579412001095

Brunner, M., Nagy, G., & Wilhelm, O. (2012). A tutorial on hierarchically structured constructs. *Journal of Personality, 80*(4), 796–846. https://doi.org/10.1111/j.1467-6494.2011.00749.x

Caspi, A., Houts, R. M., Belsky, D. W., Goldman-Mellor, S. J., Harrington, H., Israel, S., … Moffitt, T. E. (2014). The p-factor: One general psychopathology factor in the structure of psychiatric disorders? *Clinical Psychological Science, 2*(2), 119–137. https://doi.org/10.1177/2167702613497473

Caspi, A., Moffitt, T. E., Thornton, A., Freedman, D., Amell, J. W., Harrington, H. L., … Silva, P. (1996). The life history calendar: A research and clinical assessment method for collecting retrospective event-history data. *International Journal of Methods in Psychiatric Research, 6*, 101–114.

Danese, A., Moffitt, T. E., Arseneault, L., Bleiberg, B. A., Dinardo, P. B., Gandelman, S. B., … Caspi, A. (2016). The origins of cognitive deficits in victimized children: Implications for neuroscientists and clinicians. *American Journal of Psychiatry,* appi.ajp.2016.16030333. https://doi.org/10.1176/appi.ajp.2016.16030333

Dodge, K. A., Bates, J. E., & Pettit, G. S. (1990). Mechanisms in the cycle of violence. *Science*, *250*(4988), 1678+.

Finkelhor, D., Hamby, S. L., Turner, H. A., & Ormrod, R. K. (2011). *The Juvenile Victimization Questionnaire: 2nd Revision (JVQ-R2)*. Durham, NH: Crimes Against Children Research Center.

Fisher, H. L., Bunn, A., Jacobs, C., Moran, P., & Bifulco, A. (2011). Concordance between mother and offspring retrospective reports of childhood adversity. *Child Abuse & Neglect*, *35*(2), 117–122. https://doi.org/10.1016/j.chiabu.2010.10.003

Fisher, H. L., Caspi, A., Moffitt, T. E., Wertz, J., Gray, R., Newbury, J., … Arseneault, L. (2015). Measuring adolescents' exposure to victimization: The Environmental Risk (E-Risk) Longitudinal Twin Study. *Development and Psychopathology*, *27*, 1399–1416. https://doi.org/10.1017/S0954579415000838

Green, J. G., McLaughlin, K. A., Berglund, P. A., Gruber, M. J., Sampson, N. A., Zaslavsky, A. M., & Kessler, R. C. (2010). Childhood adversities and adult psychopathology in the National Comorbidity Survey Replication (NCS-R) I: Associations with first onset of DSM-IV disorders. *Archives of General Psychiatry*, *67*(2), 113. https://doi.org/10.1001/archgenpsychiatry.2009.186

Greene, A. L., & Eaton, N. R. (2017). The temporal stability of the bifactor model of comorbidity: An examination of moderated continuity pathways. *Comprehensive Psychiatry*, *72*, 74–82. https://doi.org/10.1016/j.comppsych.2016.09.010

Hamby, S., Finkelhor, D., Ormrod, D., & Turner, H. (2004). *The comprehensive JV administration and scoring manual*. Durham, NH: University of New Hampshire, Crimes Against Children Research Center.

Harmelen, A.-L. van, Tol, M.-J. van, Demenescu, L. R., Wee, N. J. A. van der, Veltman, D. J., Aleman, A., … Elzinga, B. M. (2013). Enhanced amygdala reactivity to emotional faces in adults reporting childhood emotional maltreatment. *Social Cognitive and Affective Neuroscience*, *8*(4), 362–369. https://doi.org/10.1093/scan/nss007

Herringa, R. J., Birn, R. M., Ruttle, P. L., Burghy, C. A., Stodola, D. E., Davidson, R. J., & Essex, M. J. (2013). Childhood maltreatment is associated with altered fear circuitry and increased internalizing symptoms by late adolescence. *Proceedings of the National Academy of Sciences of the United States of America*, *110*(47), 19119–19124. https://doi.org/10.1073/pnas.1310766110

Humphreys, K. L., Gleason, M. M., Drury, S. S., Miron, D., Nelson 3rd, C. A., Fox, N. A., & Zeanah, C. H. (2015). Effects of institutional rearing and foster care on psychopathology at age 12 years in Romania: follow-up of an open, randomised controlled trial. *The Lancet Psychiatry*, *2*(7), 625–634. https://doi.org/10.1016/S2215-0366(15)00095-4

Kotov, R., Krueger, R. F., Watson, D., Achenbach, T. M., Althoff, R. R., Bagby, R. M., … Zimmerman, M. (2017). The Hierarchical Taxonomy of Psychopathology (HiTOP): A dimensional alternative to traditional nosologies. *Journal of Abnormal Psychology*.https://doi.org/10.1037/abn0000258

Laceulle, O. M., Vollebergh, W. A. M., & Ormel, J. (2015). The structure of psychopathology in adolescence: replication of a general psychopathology factor in the TRAILS Study. *Clinical Psychological Science*, *3*(6), 850–860. https://doi.org/10.1177/2167702614560750

Lahey, B. B., Applegate, B., Hakes, J. K., Zald, D. H., Hariri, A. R., & Rathouz, P. J. (2012). Is there a general factor of prevalent psychopathology during adulthood? *Journal of Abnormal Psychology*, *121*(4), 971–977. https://doi.org/10.1037/a0028355

Lahey, B. B., Krueger, R. F., Rathouz, P. J., Waldman, I. D., & Zald, D. H. (2017). A hierarchical causal taxonomy of psychopathology across the life span. *Psychological Bulletin*, *143*(2), 142–186. https://doi.org/10.1037/bul0000069

Lahey, B. B., Rathouz, P. J., Keenan, K., Stepp, S. D., Loeber, R., & Hipwell, A. E. (2015). Criterion validity of the general factor of psychopathology in a prospective study of girls. *Journal of Child Psychology and Psychiatry*, *56*(4), 415–422. https://doi.org/10.1111/jcpp.12300

Lansford, J.E., Dodge, K.A., Pettit, G.S., Bates, J.E., Crozier, J., & Kaplow, J. (2002). A 12-year prospective study of the long-term effects of early child physical maltreatment on psychological, behavioral, and academic problems in adolescence. *Archives of Pediatrics & Adolescent Medicine, 156*(8), 824–830. https://doi.org/10.1001/archpedi.156.8.824

Liu, D., Diorio, J., Tannenbaum, B., Caldji, C., Francis, D., Freedman, A., … Meaney, M. J. (1997). Maternal care, hippocampal glucocorticoid receptors, and hypothalamic-pituitary-adrenal responses to stress. *Science, 277*(5332), 1659–1662. https://doi.org/10.1126/science.277.5332.1659

Magdol, L., Moffitt, T. E., Caspi, A., & Silva, P. A. (1998). Developmental antecedents of partner abuse: A prospective-longitudinal study. *Journal of Abnormal Psychology*, *107*(3), 375–389. https://doi.org/10.1037/0021-843X.107.3.375

Martel, M. M., Pan, P. M., Hoffmann, M. S., Gadelha, A., do Rosário, M. C., Mari, J. J., … Salum, G. A. (2016). A general psychopathology factor (p factor) in children: Structural

model analysis and external validation through familial risk and child global executive function. *Journal of Abnormal Psychology*. https://doi.org/10.1037/abn0000205

McLaughlin, K. A., Sheridan, M. A., Tibu, F., Fox, N. A., Zeanah, C. H., & Nelson, C. A. (2015). Causal effects of the early caregiving environment on development of stress response systems in children. *Proceedings of the National Academy of Sciences*, *112*(18), 5637–5642. https://doi.org/10.1073/pnas.1423363112

Murray, A. L., Eisner, M., & Ribeaud, D. (2016). The development of the general factor of psychopathology “p factor” through childhood and adolescence. *Journal of Abnormal Child Psychology, 44*(8), 1573–1586. https://doi.org/10.1007/s10802-016-0132-1

Muthen, L. K., & Muthen, B. O. (1998). *MPlus user’s guide* (7th ed.). Los Angeles, CA: Muthen & Muthen.

Nemeroff, C. B. (2016). Paradise Lost: the neurobiological and clinical consequences of child abuse and neglect. *Neuron, 89*(5), 892–909. https://doi.org/10.1016/j.neuron.2016.01.019

Olino, T. M., Dougherty, L. R., Bufferd, S. J., Carlson, G. A., & Klein, D. N. (2014). Testing models of psychopathology in preschool-aged children using a structured interview-based assessment. *Journal of Abnormal Child Psychology; New York*, *42*(7), 1201–11. https://doi.org/http://dx.doi.org/10.1007/s10802-014-9865-x

Patalay, P., Fonagy, P., Deighton, J., Belsky, J., Vostanis, P., & Wolpert, M. (2015). A general psychopathology factor in early adolescence. *The British Journal of Psychiatry*, *207*(1), 15–22. https://doi.org/10.1192/bjp.bp.114.149591

Rindskopf, D., & Rose, T. (1988). Some theory and applications of confirmatory second-order factor analysis. *Multivariate Behavioral Research, 23*(1), 51–67. https://doi.org/10.1207/s15327906mbr2301_3

Scott, K. M., Smith, D. R., & Ellis, P. M. (2010). Prospectively ascertained child maltreatment and its association with DSM-IV mental disorders in young adults. *Archives of General Psychiatry, 67*(7), 712–719. https://doi.org/10.1001/archgenpsychiatry.2010.71

Shakoor, S., Jaffee, S. R., Andreou, P., Bowes, L., Ambler, A. P., Caspi, A., … Arseneault, L. (2011). Mothers and children as informants of bullying victimization: Results from an epidemiological cohort of children. *Journal of Abnormal Child Psychology, 39*(3), 379–87. https://doi.org/http://dx.doi.org/10.1007/s10802-010-9463-5

Sheridan, M. A., & McLaughlin, K. A. (2014). Dimensions of early experience and neural development: deprivation and threat. *Trends in Cognitive Sciences*, *18*(11), 580–585. https://doi.org/10.1016/j.tics.2014.09.001

Snyder, H. R., Young, J. F., & Hankin, B. L. (2017). Strong homotypic continuity in common psychopathology-, internalizing-, and externalizing-specific factors over time in adolescents. *Clinical Psychological Science*, *5*(1), 98–110. https://doi.org/10.1177/2167702616651076

Straus, M. A., Murray, A., & Gelles, R. J. (1990). *Physical violence in American families: Risk factors and adaptations to violence in 8,145 families.* New Brunswick, N.J., U.S.A.: Transaction Publishers.

Teicher, M. H. (2002). Scars that won’t heal: the neurobiology of child abuse. *Scientific American*, *286*(3), 68–75.

Vachon, D. D., Krueger, R. F., Rogosch, F. A., & Cicchetti, D. (2015). Assessment of the harmful psychiatric and behavioral effects of different forms of child maltreatment. *JAMA Psychiatry*, *72*(11), 1135–1142. https://doi.org/10.1001/jamapsychiatry.2015.1792

Widom, C. S., DuMont, K., & Czaja, S. J. (2007). A prospective investigation of major depressive disorder and comorbidity in abused and neglected children grown up. *Archives of General Psychiatry*, *64*(1), 49–56. https://doi.org/10.1001/archpsyc.64.1.49

Wiesel, T. N., & Hubel, D. H. (1963). Effects of visual deprivation on morphology and physiology of cells in the cat’s lateral geniculate body. *Journal of Neurophysiology, 26*(6), 978–993.

Yung, Y.-F., Thissen, D., & McLeod, L. D. (1999). On the relationship between the higher-order factor model and the hierarchical factor model. *Psychometrika*, *64*(2), 113–128. https://doi.org/10.1007/BF02294531

Zeanah, C. H., Egger, H. L., Smyke, A. T., Nelson, C. A., Fox, N. A., Marshall, P. J., & Guthrie, D. (2009). Institutional rearing and psychiatric disorders in Romanian preschool children. *American Journal of Psychiatry, 166*(7), 777–785. https://doi.org/10.1176/appi.ajp.2009.08091438
